# Autoimmunity is a hallmark of post-COVID syndrome

**DOI:** 10.1186/s12967-022-03328-4

**Published:** 2022-03-16

**Authors:** Manuel Rojas, Yhojan Rodríguez, Yeny Acosta-Ampudia, Diana M. Monsalve, Chengsong Zhu, Quan-Zhen Li, Carolina Ramírez-Santana, Juan-Manuel Anaya

**Affiliations:** 1 Center for Autoimmune Diseases Research (CREA), School of Medicine and Health Sciences, Universidad del Rosario, Carrera 24 # 63c 69, 110010 Bogota, Colombia; 2 Clínica del Occidente, Bogota, Colombia; 3 Department of Immunology, Microarray & Immune Phenotyping Core Facility, University of Texas Southwestern Medical Center, Dallas, USA

**Keywords:** Post-COVID syndrome, Post-COVID, Long COVID, Post-acute COVID-19, COVID-19, Autoimmunity, Autoantibodies, Latent autoimmunity, Antigen array

## Abstract

Autoimmunity has emerged as a characteristic of the post-COVID syndrome (PCS), which may be related to sex. In order to further investigate the relationship between SARS-CoV-2 and autoimmunity in PCS, a clinical and serological assessment on 100 patients was done. Serum antibody profiles against self-antigens and infectious agents were evaluated by an antigen array chip for 116 IgG and 104 IgM antibodies. Thirty pre-pandemic healthy individuals were included as a control group. The median age of patients was 49 years (IQR: 37.8 to 55.3). There were 47 males. The median post-COVID time was 219 (IQR: 143 to 258) days. Latent autoimmunity and polyautoimmunity were found in 83% and 62% of patients, respectively. Three patients developed an overt autoimmune disease. IgG antibodies against IL-2, CD8B, and thyroglobulin were found in more than 10% of the patients. Other IgG autoantibodies, such as anti-interferons, were positive in 5–10% of patients. Anti-SARS-CoV-2 IgG antibodies were found in > 85% of patients and were positively correlated with autoantibodies, age, and body mass index (BMI). Few autoantibodies were influenced by age and BMI. There was no effect of gender on the over- or under-expression of autoantibodies. IgG anti-IFN-λ antibodies were associated with the persistence of respiratory symptoms. In summary, autoimmunity is characteristic of PCS, and latent autoimmunity correlates with humoral response to SARS-CoV-2.

**Supplementary Information:**

The online version contains supplementary material available at 10.1186/s12967-022-03328-4.

## Commentary

Although the majority of people with coronavirus disease 2019 (COVID-19) recover, some may experience signs and symptoms persisting or appearing after the acute illness. This condition is known as post-COVID syndrome (PCS). The specific processes behind its emergence and the impact of biological changes on clinical phenotypes remain poorly understood. Patients with PCS may exhibit persistence of inflammation and predominance of an immune effector phenotype after recovery [1].

New-onset autoantibodies have been found in acute COVID-19 [2], and latent polyautoimmunity (PolyA) seems to influence the outcomes in hospitalized patients [3]. In addition, anti-IFN antibodies are implicated in mortality and correlate with age [4]. Autoantibodies may persist after COVID-19, and latent PolyA increases over time in PCS [1]. In a recent study by Liu et al. [5], the authors observed that SARS-CoV-2 infection, even in the absence of severe clinical disease, can cause a broad autoantibody response exhibiting sex-specific patterns of prevalence and antigen specificity.

In order to further investigate the relationship between SARS-CoV-2 and autoimmunity in PCS, a clinical and serological study was conducted from March 18th to May 20th, 2021, at the Clínica del Occidente post-COVID Unit, in Bogotá, Colombia (For methods, see Additional file 1). Patients older than 18 with a history of SARS-CoV-2 infection confirmed by PCR in a swab or sputum and experiencing persistent symptoms or new symptoms after four weeks of acute illness were invited to attend the post-COVID unit. After recruitment, 100 patients were included [6]. At the time of the study, none of the patients had been vaccinated. The median age was 49 years (interquartile range—IQR: 37.8 to 55.3), and 47 were male. The median post-COVID time was 219 (IQR: 143 to 258) days. During acute COVID-19, 65 patients required hospitalization, of which 37% were admitted to the intensive care unit. Among the PCS patients, one developed polymyositis, another systemic lupus erythematosus, and an additional patient developed autoimmune thyroid disease (Additional file 2: Table S1).

**Fig. 1:**
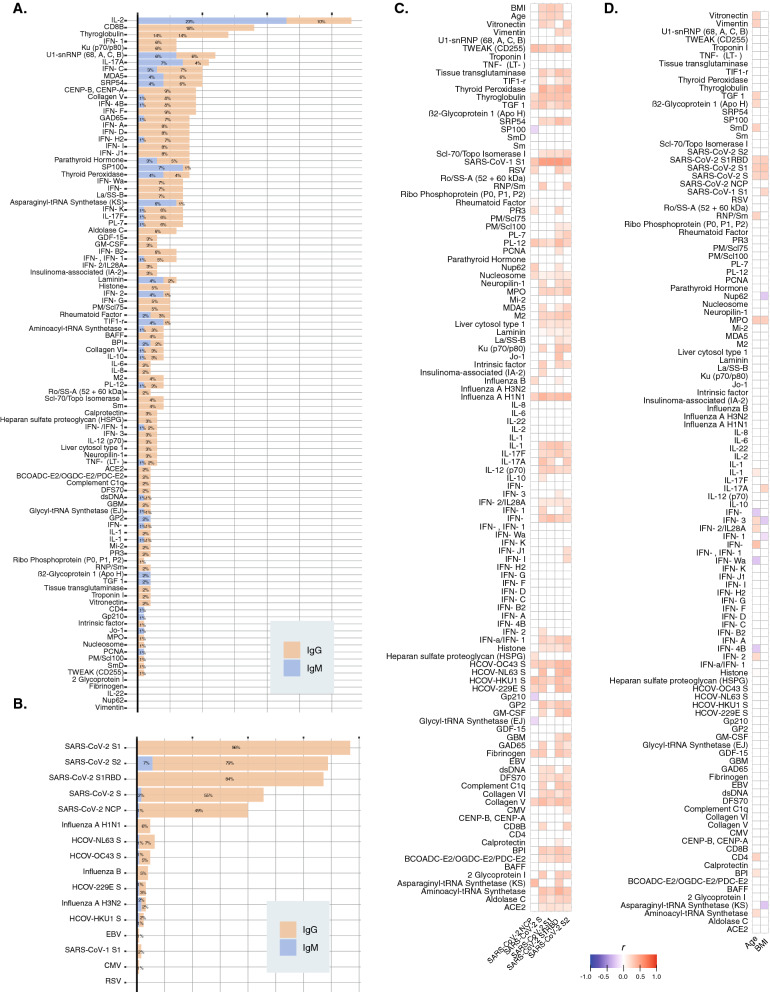
Autoimmune assessment of post-COVID syndrome. **A** Positivity of autoantibodies in patients with PCS. Antibodies were considered “positive” if NFI was > 2 SD above the average NFI for pre-pandemic controls for that antigen. **B** Positivity for antibodies against coronaviruses, influenza, Epstein-Barr, cytomegalovirus, and the respiratory syncytial virus. **C** Correlation matrix between IgG anti-SARS-CoV-2 antibodies and the rest of IgG autoantibodies included in the microarray. Blue color represents negative correlation, whereas red color represents positive correlation. Only those significant correlations by spearman tests are colored (i.e., P < 0.0500). **D** Correlation matrix between age, sex, and the IgG autoantibodies. Blue color represents negative correlation, whereas red color represents positive correlation. Only those significant correlations by spearman tests are colored (i.e., P < 0.0500). NFI: normalized fluorescence intensity

After quality control filtering of microarray, 116 IgG and 104 IgM antibodies were included in the final analysis. More than 10% of the patients were positive for IL2, CD8B, and Thyroglobulin IgG autoantibodies (Fig. 1A). Other anti-cytokine IgG autoantibodies were present in 5–10% of the patients. Overall, IgM positivity was low (Fig. 1A). Subsequent analyses only included IgG autoantibodies.

Latent autoimmunity (i.e., one IgG autoantibody) and PolyA (i.e., two or more IgG autoantibodies) were found in 83% and 62% of patients, respectively. Anti-SARS-CoV-2 IgG antibodies were found in > 85% of patients (Fig. 1B). Positivity for common cold coronaviruses, SARS − CoV − 1, and influenza was lower than 12.5% (Fig. 1B). The expression of most of the autoantibodies was correlated with IgG anti-SARS-CoV-2 antibodies against spike protein S1, S2, and RBD (Spearman correlation test, P < 0.0500) (Fig. 1C). Few autoantibodies were correlated with anti-nucleocapsid protein (NCP) IgG antibodies (Fig. 1C).

Next, we assessed the influence of age and BMI on the expression of IgG antibodies. We found that age and BMI were correlated with IgG anti-SARS-CoV-2 antibodies. However, only a few anti-IFN antibodies were correlated with age, and BMI had almost no association with any of the antibodies (Fig. 1D). Male sex was associated with IgG anti-SARS-CoV-2 antibodies for NCP (β = 1.2041, P = 0.0104) and S1 (β = 0.0239, P = 0.0239). However, the levels of remaining IgG antibodies were not affected by sex. There was no association among autoantibodies and clinical features except for anti-IFN-λ IgG antibodies associated with the persistence of respiratory symptoms (Fisher’s exact test, P = 0.0051).

Despite the high frequency of latent autoimmunity, only three patients developed overt ADs after seven months of follow-up. Su et al. [7] suggested that many autoantibodies may be present prior to the onset of the disease. However, the leap from latent autoimmunity to overt ADs may take longer [8]. Evidence indicates that latent autoimmunity may precede the appearance of autoimmune diseases several years before clinical manifestations (i.e., overt autoimmunity) [9]. Following latent autoimmunity and PolyA in patients with PCS may offer new clues on the future relevance of these latencies and the development of predictive models for autoimmunity (Table 1).

**Table 1:** Main IgG autoantibodies found in patients with post-COVID syndrome

Autoantibody	N: 100	Reported in COVID-19^a^
Anti-Tg	14 (14.0%)	Yes
Anti-CENP-B, CENP-A	9 (9.0%)	Yes
Anti-IFN-αF	9 (9.0%)	Yes
Anti-IFN-α4B	8 (8.0%)	Yes
Anti-IFN-αD	8 (8.0%)	Yes
Anti-IFN-αI	8 (8.0%)	Yes
Anti-IFN-αJ1	8 (8.0%)	Yes
Anti-GAD65	7 (7.0%)	Yes
Anti-IFN-αC	7 (7.0%)	Yes
Anti-IFN-αH2	7 (7.0%)	Yes
Anti-IFN-αWa	7 (7.0%)	Yes
Anti-IFN- ω	7 (7.0%)	Yes
Anti-La/SS-B	7 (7.0%)	Yes
Anti-IFN-αB2	6 (6.0%)	Yes
Anti-IFN-αK	6 (6.0%)	Yes
Anti- IFN-λ1	6 (6.0%)	Yes
Anti-Ku (p70/p80)	6 (6.0%)	Yes
Anti-MDA5	6 (6.0%)	Yes
Anti-PL-7	6 (6.0%)	Yes
Anti-U1-snRNP (68, A, C, B)	6 (6.0%)	Yes
Anti-Histone	5 (5.0%)	Yes
Anti-IFN-αG	5 (5.0%)	Yes
Anti-IFN-β, IFN-β1	5 (5.0%)	Yes
Anti-PM/Scl75	5 (5.0%)	Yes
Anti-CD8B	18 (18.0%)	No
Anti-IL-2	10 (10.0%)	No
Anti-Collagen V	8 (8.0%)	No
Anti-IFN-αA	8 (8.0%)	No
Anti-Aldolase C	6 (6.0%)	No
Anti-IL-17F	6 (6.0%)	No
Anti-SRP54	6 (6.0%)	No
Anti-PTH	5 (5.0%)	No

^a^ For references see Additional file 3: Table S2. There have been no descriptions for most of these autoantibodies in PCS, except for Anti-La/SS-B and Anti-U1-snRNP [7]

Although several reports, including ours [3], have shown the relevance of autoantibodies on mortality in acute COVID-19 [4], little is known about the factors associated with their emergence. Herein we confirmed that most autoantibodies are correlated with anti-SARS-CoV-2 antibodies, as shown by Liu et al*.* [5]. In patients with PCS, a proinflammatory state is evident [1], suggesting that bystander activation contributes to the emergence of autoimmunity [10]. In addition, despite the increased risk of death in patients with acute COVID-19 influenced by age, sex, and BMI [11, 12], we demonstrate that these factors do not influence the autoimmune response in PCS. It should be further characterized whether these features are implicated in new-onset overt autoimmunity.

In summary, autoimmunity is a hallmark of PCS and latent autoimmunity correlates with humoral response to SARS-CoV-2. This long-term latent autoimmune activation must be further evaluated to determine if it leads to overt autoimmunity in the future.

## Supplementary Information

**Additional file 1. **Additional Methods.

**Additional file 2: Table S1.** General characteristics of patients with overt autoimmunity during post-COVID syndrome.

**Additional file 3: Table S2.** Autoantibodies assessed in the present study.

## Data Availability

Data will be available upon request to the corresponding author.

## Abbreviations

COVID-19: Coronavirus disease 2019
NCP: Nucleocapsid protein
PCS: Post-COVID syndrome
PolyA: Polyautoimmunity
