# Supplementary material for: Autoimmunity is a hallmark of post-COVID syndrome
Source: J Transl Med. 2022 Mar 16;20:129. doi: 10.1186/s12967-022-03328-4 (PMC8924736; doi:10.1186/s12967-022-03328-4)
Supplement: Supplementary file 1 — Additional file 1. Additional Methods. [file 12967_2022_3328_MOESM1_ESM.docx]

**Additional Material**

This appendix has been provided by the authors to give readers additional information about their work.

**Additional to:** Autoimmunity is a Hallmark of Post-COVID Syndrome.

**Authors:** Manuel Rojas^1^, Yhojan Rodríguez^1, 2^, Yeny Acosta-Ampudia^1^, Diana M. Monsalve^1^, Chengsong Zhu^3^, Quan-Zhen Li^3^, Carolina Ramírez-Santana^1^, Juan-Manuel Anaya^1, 2*^

^1^ Center for Autoimmune Diseases Research (CREA), School of Medicine and Health Sciences, Universidad del Rosario, Bogota, Colombia

^2^ Clínica del Occidente, Bogota, Colombia

^3^ Department of Immunology, Microarray & Immune Phenotyping Core Facility, University of Texas Southwestern Medical Center, Dallas, USA

**Corresponding author**

*Juan-Manuel Anaya, MD, PhD.
Center for Autoimmune Diseases Research (CREA), School of Medicine and Health Sciences, Universidad del Rosario,
Carrera 24 # 63c 69, 110010 Bogota, Colombia.
E-mail address: [juan.anaya@urosario.edu.co](mailto:juan.anaya@urosario.edu.co)

**Most Recent Update:** February 07, 2022.

**Methods**

***Study design***

One-hundred patients with PCS who participated in a previous study [7], were evaluated for IgG and IgM antibodies against self-antigens,SARS-CoV-2 antigens, and other common infectious viral antigens by proteomic microarray technology [6]. A serum sample of thirty healthy pre-pandemic individuals was used as a control group for autoimmune evaluation. This study was done in compliance with Act 008430/1993 of the Ministry of Health of the Republic of Colombia, which classified it as minimal-risk research. All the patients were asked for their consent and were informed about the Colombian data protection law (1581 of 2012). The institutional review board of the Universidad CES approved the study design.

***Antibody profiling***

Serum IgG and IgM antibody reactivities against self-antigens and SARS-CoV-2 antigens was assessed with a microfluidic antigen array comprising 102 autoantigens and 16 antigens from diverse Coronaviruses, including those from SARS-CoV-2. This customized antigen panel is available from the Microarray and Immune Phenotyping Core Facility at The University of Texas Southwestern Medical Center. The various antigens are printed in duplicates on a nitrocellulose membrane-coated slide with 16 identical sub-arrays, allowing comparisons of 15 different serum samples along with a PBS control. The antigen arrays were performed as previously described [6]. In brief, serum samples were first heat-inactivated (56 °C 30 min) and pre-treated with DNAse I. Then the samples were diluted 1:100 in PBS and applied to the arrays on the slide for hybridization with the antigens. IgG and IgM antibodies binding with antigens on the array was detected with Cy-3 conjugated anti-human IgG (1/1000) and Cy5 conjugated IgM (1/1000) secondary antibodies (Jackson ImmunoResearch Laboratory). The slides were scanned with a GenePix 4400A scanner (Molecular Device) with laser wavelength 532 (for IgG) and 635 (for IgM). Images in the array were converted to Genepix Report file (GPR) with Genepix Pro7.0 software (Molecular Device). The averaged fluorescent signal intensity of each antigen was subtracted by local background and the PBS control signal and normalized to internal controls to obtain the normalized fluorescence intensity (NFI) value.

***Statistical analysis***

Univariate descriptive statistics were performed. Categorical variables were analyzed using frequencies, and quantitative continuous variables were expressed in the median and interquartile range (IQR). Data from the autoantigen array was standardized by a robust linear model as previously described [8,9]*. A*ll other parameters were analyzed without any additional data transformation. IgG and IgM antibodies were considered *“positive”* if NFI was > 2 standard deviation (SD) above the average NFI for pre-pandemic controls for that antigen.

Next, we evaluated the correlation between IgG anti-SARS-CoV-2 antibodies and the rest of antibodies included in the panel. We also explored the correlations for age and body mass index (BMI). Spearman correlation test was used in these analyses. To evaluate the influence of sex, we fitted linear regression models for each IgG autoantibody adjusted by age. The significance level of the study was set at 0.05. Statistical analyses were done using R software version 4.0.4.
