# Supplementary material for: Autoimmunity is a hallmark of post-COVID syndrome
Source: J Transl Med. 2022 Mar 16;20:129. doi: 10.1186/s12967-022-03328-4 (PMC8924736; doi:10.1186/s12967-022-03328-4)
Supplement: Supplementary file 2 — Additional file 2: Table S1. General characteristics of patients with overt autoimmunity during post-COVID syndrome. [file 12967_2022_3328_MOESM2_ESM.docx]

**Table S1.** General characteristics of patients with overt autoimmunity during post-COVID syndrome.

| **Autoimmune disease** | **Age (years)** | **Sex** | **Clinical manifestations of acute COVID-19^a^** | **Treatment of acute COVID-19** | **Clinical manifestations of autoimmune disease** | **Diagnostic test** | **Onset to autoimmunity (days)^a^** | **Positive microarray IgG antibodies** |
| --- | --- | --- | --- | --- | --- | --- | --- | --- |
| Polymyositis | 39 | M | Fever  Anosmia  Fatigue  Headache  Dry cough  Sore throat  Dizziness  Diarrhea  Emesis  Myalgia | Patient required in-hospital management.  Oxygen by cannula  prone position  corticosteroids  Antibiotics  Thromboprophylaxis | Myalgias  proximal muscle weakness | CPK: 728 U/L  Electromyography: Compatible with intrinsic muscle fiber disease, with evidence of proximal muscle involvement. | 220 | Anti-SARS-CoV-2 NCP, S1, S2, and RBD |
| Systemic lupus erythematosus | 25 | F | Anosmia  Fatigue  Headache Dizziness Confusion  Myalgia  Arthralgia  Malaise | Patient required outpatient management.  Ivermectin  Loratadine  Paracetamol  Cough syrup | Arthritis  Asthenia  Fatigue  Chest pain  Oral ulcers  Photosensitivity | Antinuclear antibodies (1/1280) homogeneous pattern (IFI)  Serum complement C4: 7.60 mg/dL.  Serum complement C3 60.90 mg/dL.  dsDNA antibodies 1/2560 (Positive).  Lupus anticoagulant ratio 1.5 (Positive).  Mild leukopenia: 3250. | 26 | BAFF, BCOADC-E2/OGDC-E2/PDC-E2  Calprotectin  CENP-B, CENP-A,  CMV, Collagen VI  dsDNA, Histone  IFN- λ2/IL28A,  IFN- λ3, Influenza B  Ku (p70/p80), Laminin  M2, Mi-2, Nucleosome  PM/Scl100, PR3,  Ro/SS-A (52 + 60 kDa), SARS-CoV-2 S1 and S2, Scl-70/Topo Isomerase I,  Sm, SP100, SRP54,  Troponin I, U1-snRNP (68, A, C, B) |
| Autoimmune thyroid disease | 58 | F | Fatigue,  Dry cough  Sore throat Dyspnea | Patient required ICU management  Antibiotic  Oxygen through high flow cannula  corticosteroid  Thromboprophylaxis | NA^b^ | Anti-thyroglobulin antibodies: 0.18 IU (Negative - ELISA).  anti-thyroid peroxidase antibodies: 101.39 IU (Positive - ELISA).  Current management with Levothyroxine. | 257^c^ | IFN- α/IFN-α1,  IFN- α2, IFN- αA,  Intrinsic factor (IF),  Anti-SARS-CoV-2 S, S1, RBD, and S2 |

^a^ Time from recovery to autoimmune disease onset.

^b^ Patient with history of non-autoimmune hypothyroidism.

^c^ Patient with prior history of hypothyroidism. During hospitalization for COVID-19, a serum sample was taken on August 29^th^, 2021. Anti-thyroid antibodies were negative. However, 257 days later, a new blood sample was taken. At this point, anti-thyroid peroxidase antibodies became positive.
