# Supplementary material for: Autoimmunity is a hallmark of post-COVID syndrome
Source: J Transl Med. 2022 Mar 16;20:129. doi: 10.1186/s12967-022-03328-4 (PMC8924736; doi:10.1186/s12967-022-03328-4)
Supplement: Supplementary file 3 — Additional file 3: Table S2. Autoantibodies assessed in the present study. [file 12967_2022_3328_MOESM3_ESM.docx]

**Table S2.** Autoantibodies assessed in the present study

| **Name** | **Abbreviation** | **Category** | **Description** | **Role in Autoimmunity** | **Evaluation in COVID-19 or Post-COVID syndrome*** |
| --- | --- | --- | --- | --- | --- |
| Interleukin-2 | IL-2 | Cytokine | IL-2 has roles in tolerance and immunity. It prevents the development of autoimmunity by promoting differentiation into regulatory T cells. In addition, IL-2 promotes differentiation into memory and effector T cells, as well as Th1 and Th2 cells. IL-2 prevents differentiation into follicular Th17 and Th cells. The main sources of IL-2 are activated CD4+ and CD8+ T cells. | Patients with systemic lupus erythematosus have a higher production of anti-IL-2 autoantibodies associated with the severity of the disease [1]. Previous studies have also demonstrated anti-IL-2 autoantibodies in healthy individuals and patients infected with the human immunodeficiency virus [2]. | Anti-IL-2 autoantibodies have not been reported in COVID-19 nor PCS. However, serum levels of this cytokine were significantly higher in patients with asymptomatic or mild COVID-19 compared with severe COVID-19 [3]. |
| CD8 Beta | CD8B | Receptor | The CD8 beta chain is a glycoprotein on the surface of cytotoxic T lymphocytes, its function is to mediate interactions between cells of the immune system. | No data | Although autoantibodies against CD8B have not been reported, the expression level of ACE2 was positively correlated with CD8A and CD8B markers on CD8+ T cells [4]. |
| Thyroglobulin | Tg | Autoimmunity | Tg is the most abundant protein in the thyroid follicles, it plays an essential role in the synthesis of thyroid hormones such as thyroxine (T4) and triiodothyronine (T3). | Tg antibodies are common in autoimmune thyroid disease, with a prevalence of 20–90% in Hashimoto thyroiditis and 30–60% in Graves' Disease. Tg antibodies are also found in autoimmune diseases such as rheumatoid arthritis, Sjögren's syndrome, systemic lupus erythematosus, and systemic sclerosis [5]. | Some case reports have described autoimmune hyperthyroidism after SARS-CoV-2 infection [6]. In addition, the development of Graves' disease (positive Tg antibodies) after vaccination with SARS-CoV-2 mRNA has been reported [7]. |
| Interferon (IFN)-λ1/ IL-29 | IFN-λ1 | Cytokine | IFN-λ1 is a member of the lambda interferon family (type III interferon), it has antiviral and immunoregulatory activity. IFN-λ1 is capable of regulating the production of IL-13 and the Th2 response. | No data | Antibodies against IFN-λ1 have not been reported. However, patients with PCS have shown a high expression of IFN-λ1 up to 8 months after SARS-CoV-2 infection [8]. Additionally, IFN-λ1 has shown an antiviral effect against SARS-CoV-2 [9]. |
| Ku (p70/p80) | Ku (p70/p80) | Autoimmunity | Ku plays important roles in multiple nuclear processes, including DNA repair, V(D)J recombination, telomere maintenance, and regulation of transcription of specific genes. | Multiple specificities of anti-Ku autoantibodies have been described in systemic lupus erythematosus and other connective tissue diseases [10]. | One case report described a patient admitted for COVID-19 pneumonia without autoimmunity on admission, who developed interstitial lung disease and anti-Ku antibodies [11]. |
| Small nuclear ribonucleoprotein complexe | U1-snRNP (68, A, C, B) | Autoimmunity | U-snRNPs are important for the splicing of mRNA precursor molecules. U1-snRNP is the most abundant RNP particle in the nucleus and consists of a small uridylate-rich RNA bound to different proteins. | Autoantibodies against U1-snRNP are present in 95% of patients with mixed connective tissue disease and in 30% of patients with systemic lupus erythematosus [12]. | Antibodies to U1-snRNP, thyroid antigens, and chromatin were observed in hospitalized COVID-19 patients [13]. In addition, a recent study reported the presence of anti-U1-snRNP antibodies in PCS [14]. |
| Interleukin 17A | IL-17A | Cytokine | IL-17A is secreted by Th17 cells and other immune cells. IL-17A is involved in the pathogenesis of various immunoinflammatory diseases including psoriasis, psoriatic arthritis, and rheumatoid arthritis. | Autoantibodies against IL-17A, IL-17F, and IL-22 have been reported in patients with chronic mucocutaneous candidiasis and autoimmune polyendocrine syndrome type I [15]. | Autoantibodies against IL-17A have not been reported in COVID-19. However, in the cytokine storm, IL-17A has played an important role in the immunopathology of COVID-19 and acute respiratory distress syndrome [16]. Recovered COVID-19 patients have elevated levels of IL-17A for up to 6 months after infection [17]. |
| Interferon-αC | IFN-αC | Cytokine | The IFNA10 gene codes for the IFN-αC protein, which belongs to the type I interferon protein family. It is an important regulatory molecule in the response to viruses and other pathogens or tumor cells. IFNA10 is associated with Th1 cell levels. | No data | Patients with COVID-19 have autoantibodies against IFN-α10 (IFN-αC) [18]. |
| Melanoma differentiation-associated protein 5 | MDA5 | Receptor | MDA5 is also known as interferon-induced helicase C domain-contacting protein I. MDA5 functions as a pattern recognition receptor capable of detecting viruses. MDA5 interacts with cellular RNA and induces an autoinflammatory response. | Anti-MDA5 antibodies have been implicated in autoimmune pathology, including dermatomyositis and rapidly progressive interstitial lung disease [19]. | Similarities between COVID-19 and anti-MDA5 syndrome have been reported [20]. Anti-MDA5 autoantibodies have been reported in 48.2% of COVID-19 patients. High titers of these autoantibodies correlated with severity [21]. |
| Signal recognition particle 54 kDa protein | SRP54 | Autoimmunity | SRP is a multimeric protein that is involved in targeting secretory proteins to the membrane of the rough endoplasmic reticulum. SRP recognizes the nascent polypeptide signal sequence on the ribosome. The SRP consists of six polypeptides (SRP9, SRP14, SRP19, SRP54, SRP68, and SRP72). | Anti-SRP54 autoantibodies occur almost exclusively in patients with polymyositis [22]. | No data |
| Centromere protein | CENP-B, CENP-A | Autoimmunity | CENP-A is a variant of histone H3, this protein determines the position of the kinetochore assembly and the final site of sister chromatid cohesion during mitosis. CENP-B is a DNA-binding protein that facilitates the assembly of specific centromere structures in interphase nuclei and mitotic chromosomes. | Autoantibodies against CENP-A and CENP-B are mainly associated with systemic sclerosis [23]. | Anti-CENPB antibodies have been reported in brain injury associated with COVID-19 [24]. |
| Collagen V | Collagen V | Autoimmunity | Type V collagen is essential for collagen fibrillation (type I and III). Additionally, type V collagen contributes to the bone matrix, corneal stroma, and interstitial matrix of muscles, liver, lungs, and placenta. | Abnormal expression of type V collagen could trigger various pathogenic events including autoimmunity, fibrosis, and vasculopathy. Anti-Collagen V autoantibodies against native and denatured protein have been reported in patients with systemic sclerosis [25]. | No data |
| Interferon-α4B | IFN-α4B | Cytokine | IFN-α4B is a subtype of interferon alpha that belongs to the IFN family of type I leukocytes. The type I IFN family has antiviral, antiproliferative, and natural killer cell activities. | Patients with autoimmune polyendocrine syndrome type 1 presented high titers of neutralizing anti-IFNα4 antibodies that are associated with a higher incidence of herpes zoster reactivation [26]. | Antibodies against IFN-α4 (IFN-α4B) have been reported in COVID-19 patients [18]. |
| Interferon-αF | IFN-αF | Cytokine | IFN-αF is a protein that in humans is encoded by the IFNA21. | No data | Antibodies against IFN-α21 (IFN-αF) have been reported in COVID-19 patients [18]. |
| Glutamic Acid Decarboxylase (65Kda) | GAD65 | Autoimmunity | GAD65 is a protein released by damaged islets. This enzyme catalyzes the production of gamma-aminobutyric acid from L-glutamic acid. | Antibodies against GAD-65 have been identified as a biomarker of autoimmune disorders of the central nervous system, as well as type 1 diabetes, autoimmune thyroid disease and pernicious anemia [27]. | Case reports have described the development of autoimmune encephalitis (positive anti-GAD65) after COVID-19 infection [28]. |
| Interferon-αA | IFN-αA | Cytokine | IFNαA is encoded by the IFNA2 gene. IFNαA plays a critical role in the acute inflammatory phase. | No data | No data |
| Interferon-αD | IFN-αD | Cytokine | IFN-αD is a protein that in humans is encoded by the IFNA1 gene. | No data | Antibodies against IFN-α1 (IFN-αD) have been reported in COVID-19 patients [18]. |
| Interferon-αH2 | IFN-αH2 | Cytokine | IFN-αH2 is encoded by the IFNA14 gene. Human IFN-α14 is the only IFN-α subtype with an N-glycosylation site. Human IFN-α14 also shows high antiviral activity against human immunodeficiency virus. | No data | Antibodies against IFN-α14 (IFN-αH2) have been reported in COVID-19 patients [18]. |
| Interferon-αI | IFN-αI | Cytokine | IFN-αI is encoded by the IFNA17 gene. | No data | Antibodies against IFN-α17 (IFN-αI) have been reported in COVID-19 patients [18]. |
| Interferon-αJ1 | IFN-αJ1 | Cytokine | IFN-αJ1 is encoded by the IFNA7 gene. | No data | Antibodies against IFN-α7 (IFN-αJ1) have been reported in COVID-19 patients [18]. |
| Parathyroid Hormone | PTH | Autoimmunity | PTH is a peptide hormone secreted by the parathyroid glands that regulates serum calcium concentration through its effects on the bones, kidneys, and intestine. | Antiparathyroid antibodies have been described in patients with idiopathic hypoparathyroidism, idiopathic Addison's disease, and Hashimoto's thyroiditis [29]. | Antibodies against PTH have not been reported. However, COVID-19 could affect the function of the parathyroid glands through direct tissue invasion by the SARS-CoV-2 [30]. |
| SP100 | SP100 | Autoimmunity | The Sp100 nuclear antigen is an interferon-stimulated antigen found in the nucleus of different human cells. | Autoantibodies directed against Sp100 have been described mainly in patients with primary biliary cirrhosis [31]. | No data |
| Thyroid Peroxidase | TPO | Autoimmunity | Thyroid peroxidase is an enzyme produced by the thyroid gland, whose function is to catalyze the oxidation of iodide and its subsequent incorporation into the thyroglobulin molecule. | TPO is a thyroid antigen that is targeted by the immune response. TPO has been associated with autoimmune hypothyroidism [32]. | Anti-TPO antibodies were detected more frequently in COVID-19 patients than in pre-pandemic controls [33]. Moreover, these autoantibodies have been also detected in patients with PCS [34]. |
| Interferon-αWa | IFN-αWa | Cytokine | IFN-αWa is encoded by the IFNA16 gene. | No data | Antibodies against IFN-α16 (IFN-αWa) have been reported in COVID-19 patients [18]. |
| Interferon-ω | IFN-ω | Cytokine | IFN-ω, although it has only one functional form described to date (IFNW1), has several pseudogenes. IFN-ω is produced by cells in response to viral infection, binds to the same receptors and activates a pathway like that activated by IFNAR. Its antigenic structure is very different from IFN-α, -β and -λ, since it does not present cross-reactivity. | High titers of IgG autoantibodies against IFN-ω have been reported in patients with myasthenia gravis [35] and autoimmune polyendocrinopathy syndrome type 1 [36]. | Auto‐Abs against IFN‐ω have been detected in critically ill patients with COVID‐19 [18,37]. |
| Single-stranded binding protein-B | La/SS-B | Autoimmunity | La/SS-B is a protein involved in various aspects of RNA metabolism. In the nucleus, La/SS-B facilitates tRNA production by facilitating folding and maturation. In the cytoplasm, La/SS-B facilitates the translation of mRNA, acting as a translation factor. La/SS-B appears to be readily removed by proteolysis, resulting in many immunoreactive polypeptides. | Anti-La/SSB antibodies can be detected in 45–60% of patients with Sjögren's syndrome [38]. | Anti-SSB antibodies have been reported in brain injury associated with COVID-19 [24]. In addition, a recent study reported the presence of anti-La/SS-B antibodies in PCS [14]. |
| Asparaginyl-tRNA Synthetase | Asparaginyl-tRNA Synthetase (KS) | Autoimmunity | Asparaginyl-tRNA synthetase (NRS) catalyzes the binding of asparagine to its cognate tRNA during translation. This enzyme catalyzes the binding of Asn and ATP to form the NRS-asparaginyl adenylate complex, followed by the esterification of Asn to its tRNA. | Anti-KS autoantibodies showed a strong association with interstitial lung disease, these autoantibodies can be found in isolation or in combination with myositis [39]. | No data |
| Interferon-αK | IFN-αK | Cytokine | IFN-αK is encoded by the IFNA6 gene. | No data | Antibodies against IFN-α6 (IFN-αK) have been reported in COVID-19 patients [18]. |
| Interleukin-17F | IL-17F | Cytokine | IL-17F is a proinflammatory signaling protein encoded by the IL17F gene. This protein is produced mainly by Th17 cells, however other epithelial and innate immune cells can produce it. IL-17F is highly (55%) homologous to IL-17A. These two molecules bind to the same receptors and most likely have similar biological functions | Autoantibodies against IL-17A, IL-17F and IL-22 have been described in patients with chronic mucocutaneous candidiasis and autoimmune polyendocrine syndrome type I [15]. | Autoantibodies against IL-17F have not been reported in COVID-19. IL-17F along with other cytokines plays an important role in the immunopathology of COVID-19 [16]. |
| Threonyl t-RNA Synthetase | PL-7 | Autoimmunity | Threonyl-tRNA synthetase is a member of the aminoacyl-tRNA synthetase family, these enzymes are key to protein biosynthesis. | Anti-PL-7 antibodies have been detected in patients with polymyositis and dermatomyositis. Antisynthetase syndrome is characterized by the presence of antisynthetase autoantibodies [40]. | A patient with COVID-19 and anti-PL7 autoantibodies has been reported [41]. |
| Aldolase C | ALDOC, or ALDC | Autoimmunity | Aldolase C, fructose bisphosphate (ALDOC or ALDC) is an enzyme encoded by the ALDOC gene. ALDOC is a key enzyme in glycolysis and gluconeogenesis, it catalyzes the reversible conversion of fructose-1,6-bisphosphate to glyceraldehyde-3-phosphate or glyceraldehyde and dihydroxyacetone phosphate. ALDOC participates in the stress response pathway generated by hypoxia in lung epithelial cells. | Aldose has been identified in sera from patients with Alzheimer's disease as the major autoantigen [42]. Additionally, the presence of antinuclear antibodies, rheumatoid factor and aldolase were associated with interstitial lung disease [43]. | No data |
| Growth/differentiation factor 15 | GDF-15 | Cytokine | GDF15 was first identified as macrophage inhibitory cytokine-1 or MIC-1. It is a protein belonging to the transforming growth factor beta superfamily. GDF-15 plays a role in the regulation of inflammatory pathways and is involved in the regulation of apoptosis, cell repair, and cell growth. | No data | Anti-GDF-15 autoantibodies have not been reported in COVID-19 or PCS. However, the GDF-15 protein appears to be a strong predictor of poor outcomes in critically ill patients with COVID-19 [44]. |
| Granulocyte Macrophage Colony-Stimulating Factor | GM-CSF | Cytokine | GM-CSF is a pleiotropic cytokine whose function is to regulate the proliferation and differentiation of myeloid precursor cells, granulocytes, and phagocytes. | Anti-GM-CSF autoantibodies have been associated with some cases of cryptococcal meningitis in immunocompetent patients [45]. In addition, anti-GM-CSF autoantibodies have been reported in autoimmune pulmonary alveolar proteinosis [45]. | Anti-GM-CSF autoantibodies have not been reported in COVID-19 or PCS. However, high levels of GM-CSF have been described in patients with severe COVID-19 [46]. |
| Interferon alpha B2 | IFN-αB2 | Cytokine | This is a subtype cytokine member of the type I interferon family that is produced in response to viral infection as a part of the innate immune response. Predicted to be involved in several processes, including lymphocyte activation involved in immune response and positive regulation of peptidyl-serine phosphorylation of STAT protein. IFN-α8 (IFN-αB2) displays a strong capacity to induce IFIT1, CXCL10, CXCL11, ISG15 and CCL8, and is a highly potent inducer of primary B cell proliferation | Autoantibodies against IFN-α2, IFN-α8, IFN-ω, and IL-12 have been observed in autoimmune Polyendocrinopathy Syndrome Type 1 Patients [36]. | Bastard et al.[18] showed that all patients tested with autoantibodies against IFN-α2 also had autoantibodies against all 13 IFN-α subtypes, among them anti-IFNα8 (IFN-αB2). |
| Interferon beta 1 | IFN-β/IFN-β1 | Cytokine | Cytokine that belongs to the type I interferon family, which are released as part of the innate immune response to pathogens. IFN-β is naturally produced by fibroblasts. Following secretion in response to a pathogen, type I interferons bind a homologous receptor complex and induce transcription of genes such as those encoding inflammatory cytokines and chemokines. | Diseases associated with IFNB1 include primary progressive multiple sclerosis and relapsing-remitting multiple sclerosis. IFN-β is being used to treat certain autoimmune and inflammatory diseases, such as relapsing-remitting multiple sclerosis familiar Mediterranean fever, and Behcet's syndrome [47]. | Studies have showed that patients with critical COVID-19 or deceased patients can have IFN-β autoantibodies [18,48]. Antibodies against IFN-β have not been reported in PCS. However, patients with PCS showed elevated expression of IFN-β that remained persistently high at 8 months after infection [8]. |
| Interferon Lambda 2/Interleukin-28A | IFN-λ2/IL28A | Cytokine | Type III IFNs (IFN-λ -1, -2 and -3) were discovered as IL-29, 28A and 28B and have many immune activities in common with type I IFNs. The IFN-λs have been demonstrated to be induced after stimulation with several single-stranded RNA (ssRNA) viruses. This cytokine plays a critical role in the antiviral host defense, predominantly in the epithelial tissues. Acts as a ligand for class II cytokine receptor composed of IL10RB and IFNLR1, resulting in the expression of IFN-stimulated genes (ISG), which mediate the antiviral state. | Several studies found higher serum concentrations of type III IFNs in patients with systemic lupus erythematosus when compared to healthy controls and even higher concentrations in patients with active systemic lupus erythematosus. Type III IFN levels correlated with systemic lupus erythematosus disease activity index scores and dsDNA autoantibody titers [49,50]. | Wang et al.[51] performed a principal component analysis in patients with COVID-19 and found the second component comprised autoantibody reactivity against cytokines, chemokines and the type III IFNs (IFNλ2 and IFNλ3). Antibodies against IFN-β have not been reported in PCS. However, there are different combinations of the inflammatory mediators IFN-λ2/3, IFN-β, PTX3, IFN-γ and IL-6 that are associated with PCS [8]. |
| Insulinoma-associated protein-2 | IA-2 | Autoimmunity | IA-2 is an enzymatically inactive transmembrane glycoprotein localized in the dense core secretory granules of peptide-secreting endocrine cells and neurons. Together with GAD65 and insulin, the islet antigen IA-2 is a major autoantigen in type 1 diabetes that occurs through autoimmune-mediated beta-cell destruction. Within the cytoplasmic region of IA-2, the PTP-like domain is the major region for autoantibody recognition, and the juxtamembrane region has a few immune recognition epitopes for the autoantibodies found early in the disease. | Major autoantigen in type 1 diabetes, about 60% of new-onset type 1 diabetic patients produce IA-2 autoantibodies and that these autoantibodies may appear years before the onset of clinical disease, thus being a useful marker to identify subjects at high risk for developing type 1 diabetes [52,53]. | No data |
| Laminin | Laminin | Autoimmunity | Laminins are a major constituent of the basement membrane which is an intricate meshwork of proteins separating the epithelium, mesothelium, and endothelium. Laminins are heterotrimeric glycoproteins, composed of α, β, and γ chains. Self-polymerization of laminins is a critical step in basement membrane formation, and is dependent on cell membrane interactions with integrins, dystroglycan, the lutheran glycoprotein, and sulfated glycolipids. The laminins are an important and biologically active part of the basal lamina, influencing cell differentiation, migration, and adhesion. | Detection of antibodies directed at the basement membrane of the skin led to the identification of a subgroup of patients with pemphigoid who had serum antibodies reactive against laminins. Laminin γ1 was reported as an antigen in anti-p200 pemphigoid and anti-laminin γ1 antibodies were further linked to collagenases [54]. Autoimmunity against laminins is associated mainly with mucous membrane pemphigoid and systemic lupus erythematosus [55]. | Autoantibodies against laminin γ have been observed in patients with brain injury in COVID-19 [55]. |
| Histone | Histone | Autoimmunity | Histones are a family of basic proteins, and their positive charges allow them to associate with DNA in the nucleus and help condense it into chromatin creating structural units called nucleosomes. Histone variants can modulate the highly dynamic nature of the nucleosome to accommodate for different nuclear DNA metabolic needs such as transcription, replication, and repair. | Anti-histone antibodies occur in several autoimmune diseases, including systemic lupus erythematosus, drug-induced lupus, juvenile rheumatoid arthritis and rheumatoid arthritis. The frequency of autoantibodies to individual histones vary according to the type of autoimmune disease. In general, antibodies to H1 are the most frequent in systemic lupus erythematosus, followed by anti-H2B, anti-H2A, anti-H3 and anti-H4 [56]. | Pascolini et al.[57] showed COVID‐19 patients with reactivity to at least one autoantibody. Specifically, 33.3% of the patients had ANA reactivity, characterized in one patient by anti‐histone antibodies on specific ENAs. |
| Interferon Alpha 2 | IFN-α2 | Cytokine | This cytokine is a member of the type I interferon family that is produced in response to viral infection as a key part of the innate immune response with potent antiviral, antiproliferative and immunomodulatory properties. This cytokine, like other type I interferons, binds a plasma membrane receptor made of IFNAR1 and IFNAR2 that is ubiquitously expressed, and thus is able to act on virtually all body cells. A deficiency of type I interferon in the blood is thought to be a hallmark of severe COVID-19 and may provide a rationale for a combined therapeutic approach. | Antibodies against interferon-α2 and/or interferon-ω have been detected in autoimmune polyendocrine syndrome [58]. In addition, a sustained increase in IFN-I may accompany clinical manifestations and disease activity in systemic autoimmune diseases [59]. | Bastard et al.[18] reported that patients with life-threatening COVID-19 pneumonia had autoantibodies against IFN-ω, against the 13 types of IFN-α (between them anti-IFN-α2), or against both at the onset of critical disease. These autoantibodies neutralize the ability of the corresponding type I IFNs to block SARS-CoV-2 infection in vitro. Other study detected IFN-α2 antibodies in COVID-19 patients, but not in ICU patients with other respiratory illnesses. The presence of neutralizing IFN-α2 antibodies in critically ill COVID-19 was associated with delayed viral clearance [60]. A recent study reported the presence of anti-IFN-α2 antibodies in PCS [14]. |
| Interferon Alpha 5/G | IFN-αG | Cytokine | Natural human leukocyte α-interferon is a mixture of at least 10 IFN-α species. Minor species include IFN-α5 (IFN-αG), IFN-α14 and IFN-α16. IFN-α5 has been found to be the main IFN-α subtype expressed in the liver of healthy people and is markedly decreased in the livers of patients with chronic HCV infection compared with patients with other liver diseases (including hepatitis B virus chronic infection). It has also been demonstrated that IFN-α5 induces stronger signaling and higher expression of antiviral genes than IFN-α2. | The type I IFN pathway is prominently upregulated in patients with several autoimmune diseases such as systemic lupus erythematosus, dermatomyositis, Sjögren's syndrome, systemic sclerosis and type I interferonopathies [61]. | Bastard et al.[18] showed that all patients tested with autoantibodies against IFN-α2 also had autoantibodies against all 13 IFN-α subtypes, among them anti-IFNα5 (IFN-αG). |
| polymyositis/scleroderma complex | PM/Scl75 | Autoimmunity | PM-Scl complex was shown to be the human equivalent of the yeast exosome, a complex consisting of at least 10 proteins, all displaying characteristics of exoribonucleases, which has been shown to be involved in the degradation and processing of many different RNA species. The primary target antigens of PM-Scl antibodies are the proteins PM-Scl 100 (100 kDa) and PM-Scl 75 (75 kDa). PM-Scl 100 is detected virtually by 100 % of PM-Scl antibodies, and PM-Scl 75 by 50-60 %. | Autoantibodies against the PM/Scl complex are found in ~24% of patients with polymyositis/scleroderma overlap syndrome, compared with only 2% of patients with scleroderma alone and 6% of patients with myositis (polymyositis/dermatomyositis) alone. In contrast, between 43% and 88% of patients positive for anti-PM/Scl antibodies are diagnosed with myositis/scleroderma overlap syndrome [62,63]. | Case report of a case of mild COVID-19 infection complicated by autoantibody production (Anti-PM/Scl 75 and PM/Scl 100 resulted positive) and cutaneous and gastrointestinal symptoms and subsequently diagnosed with systemic sclerosis. This case describes that SARS-CoV-2 may trigger systemic sclerosis [64]. |
| Rheumatoid Factor | RF | Autoimmunity | Rheumatoid factors are antibodies with various isotypes and affinities, directed against the Fc portion of IgG. The commonly mentioned RF is an IgM, although other immunoglobulin types, including IgG and IgA, are rarely found. RFs can be found in the serum of patients with a wide range of autoimmune and inflammatory diseases as well as in normal individuals, especially with age. | RFs occur in approximately 60 to 80% of patients with rheumatoid arthritis and represent one criterion for the classification or diagnosis of this disease. These autoantibodies are found not only in rheumatoid arthritis but in a wide range of pathologies including other autoimmune and non-autoimmune diseases. They have been found in up to 4% of young, healthy individuals and the elderly as well. Moreover, patients with rheumatic diseases like Sjögren's syndrome, mixed connective tissue diseases, mixed cryoglobulinemia, and systemic lupus erythematosus have elevated RFs [65]. | Anaya et al.[33] showed that hospitalized patients with COVID-19 had latent autoimmunity characterized by a high frequency of anti-TPO, RF, anti-CCP3, antinuclear antibodies, IgM anti-Beta-2 glycoprotein-1 and IgM anti-cardiolipin antibodies. A meta-analysis confirmed these results, with RF and antinuclear antibodies being the most common autoantibodies. In addition, several studies have showed the development of rheumatoid arthritis during or after of SARS-CoV-2 infection with presence of RF antibodies [66–68]. |
| Transcription intermediary factor 1 | TIF1 | Autoimmunity | TIF1 is a protein belonging to the tripartite motif (TRIM) super family and is found in four subtypes, known as TIF1-α (TRIM24), TIF1-β(TRIM28), TIF1-γ(TRIM33), and TIF1-δ(TRIM66). TRIM is an ubiquitin ligase involved in protein modification and the C-terminal chromatin binding unit performs epigenetic transcriptional regulation. TIF1-γ has been reported to play a role in transcriptional elongation, DNA repair, differentiation of cells, embryonic development, and mitosis. | Myositis-specific autoantibodies (MSAs) can be identified in 60–70% of patients with idiopathic inflammatory myopathies. Of these MSAs, anti-TIF1-γ, anti-NXP2 and anti-HMGCR autoantibodies have been reported to be associated with an increased risk of cancer in patients with idiopathic inflammatory myopathies [69,70]. TIF1-γ, has proven useful for cancer screening in patients with dermatomyositis [71]. | A study by Megremis and colleague [72] identified three immunogenic linear epitopes with high sequence identity to SARS-CoV-2 proteins in anti-TIF1-γ dermatomyositis, which it can suggest the presence of an overlapping mechanism between COVID-19 and dermatomyositis. |
| Aminoacyl-tRNA Synthetase | ARSs | Autoimmunity | These are a family of 20 essential enzymes (one for each amino acid) that ligate amino acids to their corresponding tRNAs in protein synthesis. Each ARS protein and the encoding gene are designated as XRS and XARS, respectively, where X designates the single-letter amino acid code of the cognate amino acid. For example, KRS designates the lysyl-tRNA synthetase and KARS is the gene encoding it. ARSs can be grouped into class I and class II, which are characterized by fundamental structural differences and enzyme kinetics. | Autoantibodies against ARSs were found in anti-synthetase syndrome, suggesting that ARSs are likely to be involved in the development and progression of autoimmune diseases [73]. Autoantibodies to six of the ARSs (Jo1, PL7, PL12, OJ, EJ, and KS) have been described in patients with connective tissue diseases, and these autoantibodies can be found in patients with inflammatory muscle disorders, polymyositis and dermatomyositis [74]. | No data |
| B-cell-activating factor (TNFSF13B) | BAFF | Cytokine | B-cell-activating factor (BAFF) is a member of the tumor-necrosis factor family. This cytokine is a ligand for receptors TNFRSF13B/TACI, TNFRSF17/BCMA, and TNFRSF13C/BAFFR. This cytokine is expressed in B cell lineage cells, and acts as a potent B cell activator. It has been also shown to play an important role in the proliferation and differentiation of B cells. | Elevated serum and salivary gland levels of BAFF have been detected in patients with Sjögren's syndrome. In addition, BAFF levels in autoimmune diseases, such as multiple sclerosis, systemic lupus erythematosus, and rheumatoid arthritis, were found to be significantly increased [75,76]. A study demonstrated a high prevalence of antibodies anti-BAFF in a multi‐ethnic Asian systemic lupus erythematosus cohort. While levels of serum BAFF correlated positively with disease activity in these patients, levels of anti‐BAFF antibodies were correlated negatively with levels of its target cytokine, anti‐dsDNA antibodies and clinical disease activity [77]. | Antibodies against BAFF have not been reported. However, in COVID-19, neutrophil counts are increased and IFN stimulation is part of the inflammatory reaction. Schultheiß et al.[78] observed a positive correlation between BAFF levels and B cell count in COVID-19, it seems plausible that B cell counts are driven by neutrophil granulocyte-produced BAFF. Another study showed that BAFF, IL-18, IL-2, and IL-4 may be beneficial for the recovery of COVID-19 patients [79]. |
| Bactericidal permeability-increasing protein | BPI | Autoimmunity | BPI belongs to family of lipid-binding serum glycoproteins. It is associated with human neutrophil granules and has antimicrobial activity against gram-negative organisms. Also it is found in other tissues including the epithelial lining of mucous membranes. The cytotoxic action of BPI is limited to many species of Gram-negative bacteria; this specificity may be explained by a strong affinity of the very basic N-terminal half for the negatively charged lipopolysaccharides that are unique to the Gram-negative bacterial outer envelope. | Anti-BPI autoantibodies have been found in a variety of clinical settings such as cystic fibrosis, inflammatory bowel diseases, TAP deficiency and reactive arthritis. A common feature of these conditions is the chronic exposure of the host to Gram-negative bacteria and their endotoxin [80]. | No data |
| Collagen VI | Collagen type VI | Autoimmunity | Type VI collagen is a unique beaded filament collagen and is found in the interface between the basement membrane and interstitial matrix, where it forms a unique microfibrillar network. Collagen VI, a ubiquitously expressed extracellular matrix protein, forms microfibrils in close association with the basal lamina around the muscle fibers. This maintains a mechanical function in the cell, which is typical of most types of Collagen, by providing stability and structural support in the Extracellular matrix. Collagen type VI is a microfibrillar collagen found in many extracellular matrices including those of muscle, skin, tendon, and vessels [81]. | It is not clear what triggers the inflammatory response in Ulrich congenital muscular dystrophy but over-expression of MHC-class I components suggest an autoimmune component which may be directed towards the abnormal collagen VI which is produced by these patients. | No data |
| Interleukin-10 | IL-10 | Cytokine | Major immune regulatory cytokine that acts on many cells of the immune system where it has profound anti-inflammatory functions, limiting excessive tissue disruption caused by inflammation. IL-10 is a cytokine produced primarily by monocytes and to a lesser extent by lymphocytes. This cytokine has pleiotropic effects in immunoregulation and inflammation. It down-regulates the expression of Th1 cytokines, MHC class II antigens, and costimulatory molecules on macrophages. It also enhances B cell survival, proliferation, and antibody production. This cytokine can block NF-kappa B activity and is involved in the regulation of the JAK-STAT signaling pathway. | IL-10 plays an important role in both the onset and development of autoimmune diseases, such as systemic lupus erythematosus, rheumatoid arthritis, Sjögren's syndrome, multiple sclerosis, Crohn’s disease, and psoriasis. IL-10 affects the immune system and influences many pathophysiological processes through the regulation of cytokines and growth factors. IL-10 strengthens peripheral tolerance of regulatory T cells and it is related with activation and apoptosis of B cell and regulation of antigen presentation, participating in the regulation of inflammation and autoantibody production [82]. | Antibodies against IL-10 have not been reported. IL-10 was thought to be a negative feedback mechanism to suppress inflammation. However, several lines of clinical evidence suggest that dramatic early proinflammatory IL-10 elevation may play a pathological role in COVID-19 severity [83]. Another study showed that COVID-19 patients had higher serum level of cytokines (TNF-α, IFN-γ, IL-2, IL-4, IL-6 and IL-10). Specifically, IL-6 and IL-10 levels were significantly higher in critical group becoming predictors of severe diseases [84]. |
| Interleukin-6 | IL-6 | Cytokine | IL6 is a potent inducer of the acute phase response. Rapid production of IL6 contributes to host defense during infection and tissue injury, but excessive IL6 synthesis is involved in disease pathology. In the innate immune response, is synthesized by myeloid cells, such as macrophages and dendritic cells, upon recognition of pathogens through TLRs at the site of infection or tissue injury. In the adaptive immune response, is required for the differentiation of B cells into immunoglobulin-secreting cells. Plays a major role in the differentiation of CD4 (+) T cell subsets. Essential factor for the development of T follicular helper (Tfh) cells that are required for the induction of germinal-center formation. Required to drive naive CD4(+) T cells to the Th17 lineage. In addition, the encoded protein has been shown to be an endogenous pyrogen capable of inducing fever in people with autoimmune diseases or infections. | A variety of studies have demonstrated that overproduction of IL-6 contributes to the pathogenesis of various autoimmune and inflammatory diseases. Increased IL-6 levels are observed in RA, systemic-onset juvenile chronic arthritis, osteoporosis, and psoriasis [85]. Moreover, IL-6 is required for experimentally induced autoimmune diseases, including type II collagen-induced arthritis and antigen-induced arthritis, myelin oligodendrocyte protein-induced experimental autoimmune encephalomyelitis and pristane-induced autoantibody production [86]. | At the start of the pandemic, elevated cytokine levels (IL-6, GM-CSF, TNF, interferons) were commonly reported in severely ill patients with COVID-19. These cytokines were often portrayed as part of a dysregulated host response to SARS-CoV-2 that promoted severe disease [87]. IL-6 behaves as a better predictor of disease progression. In addition, IL-6 level was the most significant predictor of the non-survivors group in acute COVID [88]. On the other hand, a proinflammatory state was observed in patients with PCS characterized by upregulated IL-6, IFN-α, TNF-α, G-CSF, IL-17A, IL-1β, and IL-13 [89]. High concentrations of IL-6 may contribute to fatigue, sleeping difficulties, depression, and anxiety suggesting that a maintained inflammation is associated with PCS [90,91]. |
| Interleukin-8 | IL-8 | Cytokine | IL-8 is secreted by mononuclear macrophages, neutrophils, eosinophils, T lymphocytes, epithelial cells, and fibroblasts. It functions as a chemotactic factor by guiding the neutrophils to the site of infection. Also, IL-8 is a chemotactic factor that attracts basophils, and T-cells, but not monocytes. Bacterial and viral products rapidly induce IL-8 expression. IL-8 also participates with other cytokines in the proinflammatory signaling cascade and plays a role in systemic inflammatory response syndrome. | IL-8 is key to recruitment of polymorphonuclear leukocytes to the intestinal mucosa of patients affected with Crohn's disease or ulcerative colitis. Its levels are elevated in lesions from both types of inflammatory bowel disease [92,93]. Moreover, IL-8 is increased in the serum of very early rheumatoid arthritis patients and in synovial fluid of established rheumatoid arthritis [94]. | Antibodies against IL-8 have not been reported. However, Li et al [95] showed that both IL-6 and IL-8 serum levels were elevated in COVID-19 patients with severe diseases showing that IL-8 levels correlated better than IL-6 with the overall clinical disease scores at the different time points in the same COVID-19 patients, which can therefore be used as a disease prognosis biomarker. Another study showed an association between serum levels of IL-8 and the duration of illness in patients with severe COVID-19 [96]. Moreover, IL-8 levels were associated with in-hospital death in severe to critical COVID-19 patients, which could help to identify patients with high risk of death [97]. |
| Mitochondrial protein 2 | M2 | Autoimmunity | M2 is a cluster of four major mitochondrial inner membrane proteins of approximately 74, 52, 45 and 39 kDa. Contains the E2 subunit of branched chain 2-oxo acid dehydrogenase, the E2 subunit of the 2-oxo dehydrogenase complex, protein X and the E1a and E1b subunits of the pyruvate dehydrogenase complex. | Anti-mitochondrial M2 antibody (AMA-M2) is specific to primary biliary cirrhosis, but can also be found in certain patients with autoimmune hepatitis [98]. In addition, antibodies anti-mitochondrial were observed in human and murine systemic lupus. mitochondrial antibodies in human sera showed an association with anti-dsDNA as well as with lupus nephritis. Detection of antibodies to mitochondrial components in systemic lupus erythematosus may provide novel information on patients, such as their risk for developing nephritis [99]. | A case of primary biliary cholangitis developing during or soon after COVID-19 has been reported. The patient was tested positive for ANA and AMA (M2/BCOADC) at high titer. A liver biopsy was performed and showed florid ductal lesions, therefore leading to the definite diagnosis of PBC. This was the first reported case of primary biliary cholangitis induced by SARS-CoV-2 [100]. |
| Alanyl t-RNA synthetase (PL 12) | PL-12 | Autoimmunity | The human alanyl-tRNA synthetase belongs to a family of tRNA synthases, of the class II enzymes. During mRNA translation at ribosomes, specific amino-acyl-tRNA synthetases are required for the covalently attachment between tRNAs and different amino acids. Alanyl -tRNA synthetase (AARS, PL-12) is specific for the amino acid alanine. | PL-12 is recognized by PL-12 autoantibodies, which is present in a subset of patients with polymyositis and dermatomyositis [101]. | No data |
| Sjögren's-syndrome-related antigen A | Ro/SS-A (52 + 60 kDa) | Autoimmunity | Ro is a member of the tripartite motif (TRIM) family. Ro antigens were found to consist of two different proteins, Ro60 and Ro52. Studies provided evidence that Ro52 and Ro60 are localized to different cell compartments and that anti-Ro52 and anti-Ro60 antibodies have different clinical associations. Ro52 is an interferon (IFN)-inducible protein, and it is induced by viral infection or TLR engagement via type I IFN induction. Following the first demonstration of Ro52 ubiquitin E3 ligase activity by Wada et al. several reports supporting the conclusion have been published by other groups. In addition, Ro52 is a negative regulator for proinflammatory cytokine production. Ro60 protein, having a ring shape, binds to misfolded noncoding RNAs in vertebrate nuclei and acts as a quality checkpoint for RNA misfolding with molecular chaperones for defective RNAs. The misfolded RNAs are targeted by Ro60 for degradation. | Anti-Ro/SSA antibodies are among the most frequently detected autoantibodies against extractable nuclear antigens and have been associated with systemic lupus erythematosus and Sjögren's syndrome. They are also sometimes seen in other systemic autoimmune diseases, such as systemic sclerosis, polymyositis/dermatomyositis, mixed connective tissue disease, and rheumatoid arthritis [102]. | Fujii et al.[103] observed an elevation of anti-SSA/Ro antibodies suggesting a contribution from autoimmunity functions. Although it is unclear whether the elevation of anti-SSA/Ro antibodies was a cause or an outcome of aggravated COVID-19 pneumonia, they hypothesize that both patients developed aggravated the COVID-19 pneumonia due to an autoimmune response. Several studies have reported the presence of autoantibodies in patients with COVID-19: antinuclear antibodies in 35.6%, anti-Ro/SSA in 25%, RF in 19%, lupus anticoagulant in 11% and antibodies against IFN-I in 10% showing that autoimmune phenomena exist in COVID-19 subjects [104,105]. A recent study reported the presence of anti-Ro/SS-A antibodies in PCS [14]. |
| Scl-70/Topo Isomerase I | Scleroma 70-kD | Autoimmunity | DNA topoisomerases are able to solve topological problems resulting from replication, transcription, recombination, and reorganization of the chromatin. Further on, topoisomerases change the state of supercoiling of the DNA and therefore, have great impact on gene activity. In order to decrease gene activity, DNA topoisomerases introduce temporary single-strand breaks (type I) or double-strand breaks (type II) in the phosphate backbone of the DNA. The mechanism of topoisomerase action includes the transient formation of an ester bond between a tyrosine residue of the enzyme and the DNA molecule. Later on, the breaks are closed by reformation of the original phosphodiester-bond and the enzyme released from the DNA. | Anti–Scl-70 antibodies are considered a specific marker for the diffuse type of systemic sclerosis. Although found in approximately 20% of systemic sclerosis sera, other evidence indicated that anti-Scl-70 also occur in up to 25% of systemic lupus erythematosus [106]. Moreover, patients with autoimmune hypothyroidism have high levels of anti-Scl-70 levels in comparision to controls. Anti-TPO, anti-Tg and TSH significantly correlated with anti-Scl-70 [107]. | anti-PM-Scl or anti-Scl-70 antibodies were detected in COVID-19 patients with specific ENAs who developed acute respiratory distress syndrome. Of note, a significant proportion of anti-PM-Scl-/anti-Scl-70 positive patients develop pulmonary fibrosis, raising the question of the long-term effects of severe COVID-19 in these patients [108]. |
| Sm | Smith antigen | Autoimmunity | Sm antigen is a non-histone nuclear protein composed of several polypeptides of differing molecular weights. The various kinds of Sm rings function as scaffolds or chaperones for RNA oligonucleotides, assisting the RNA to assume and maintain the proper three-dimensional structure. In some cases, this allows the oligonucleotide RNA to function catalytically as a ribozyme. In other cases, this facilitates modification or degradation of the RNA, or the assembly, storage, and intracellular transport of ribonucleoprotein complexes. | Among autoantibodies against ENA, anti-dsDNA antibodies and anti-Sm antibodies are highly specific for systemic lupus erythematosus, and the presence of anti-dsDNA and/or anti-Sm antibodies is one of the important criteria for the classification of systemic lupus erythematosus. The specificity of anti-Sm antibodies for the classification of systemic lupus erythematosus reached 90% [109]. Some studies have suggested that patients with anti-Sm antibodies are more likely to have renal involvement and central nervous system dysfunction [110]. | No data |
| Calprotectin | CLP | Autoimmunity | CLP, a calcium-binding protein of the S100 family, performs various biological functions via interaction with TLR4 on the surface of leukocytes and is manly released by activated neutrophils, monocytes and early differentiation states of macrophages. CLP has been shown to perform various biological functions, especially in triggering signaling pathways involved in inflammatory processes and inhibition of microbial growth. The presence of calprotectin in feces is a consequence of neutrophil migration into the gastrointestinal tissue due to an inflammatory process. | Serum CLP can be used as a potential biomarker for myasthenia gravis [111]. In inflammatory bowels diseases levels of CLP were significantly increased in patients with active disease and strongly predicted disease relapse and treatment response [112]. In addition, CLP is found in other autoimmune diseases such as rheumatoid arthritis. Specifically, CLP (S100A8 and S100A9) are the most up-regulated proteins in rheumatoid arthritis synovial tissue and synovial fluid [113]. | Antibodies against CLP have not been reported. However, a study showed that fecal and serum CLP levels were not significantly different between COVID-19 patients with gastrointestinal symptoms and those without. However, both fecal and serum calprotectin were found to be superior in identifying COVID-19 patients who progressed to severe disease [114]. Moreover, in COVID-19 patients, CLP levels were associated with poor clinical outcomes, such as significantly reduced survival time, especially in patients with severe lung disease [115]. Serum CLP levels have been shown to strongly track the current and eventual severity of COVID-19, strongly implicating neutrophils as active perpetuators of inflammation and respiratory compromise in COVID-19. In addition, CLP levels are also elevated in those patients who required mechanical ventilation during their hospitalization [116]. |
| Heparan sulfate proteoglycan | HSPG | Autoimmunity | HSPGs are glycoproteins, with the common characteristic of containing one or more covalently attached heparan sulfate (HS) chains, a type of glycosaminoglycan (GAG). HSPGs are present in basement membranes (perlecan, agrin, and collagen XVIII), where they collaborate with other matrix components to define basement membrane structure and to provide a matrix for cell migration. HSPGs can bind cytokines, chemokines, growth factors, and morphogens, protecting them against proteolysis. | Autoimmunity to HSPG has been demonstrated to play a role in vascular injury in animal models, and is present in patients with autoimmune vascular disease and systemic lupus erythematosus [117]. | Antibodies against HSPG have not been reported. However, the S protein of SARS‐CoV‐2 interacts with heparan sulfates, which might be required for infection [118]. The cell surface HS can serve as an anchor point to facilitate endocytosis of many cargos, which include SARS-CoV-2, related coronaviruses. HS as an attachment factor that assists SARS coronavirus cell entry [119]. In addition, a study showed that mucosal dendritic cell (DC) subsets captured SARS‐CoV‐2 via heparan sulfate proteoglycans. The different DC subsets were not infected but transmitted SARS-CoV-2 to ACE2-positive cells, which could facilitate virus dissemination [120]. |
| Interferon Alpha 1 | IFN-α/IFN-α1 | Cytokine | This cytokine is a member of the type I interferon family that is produced in response to viral infection as a key part of the innate immune response with potent antiviral, antiproliferative and immunomodulatory properties. This cytokine, like other type I interferons, binds a plasma membrane receptor made of IFNAR1 and IFNAR2 that is ubiquitously expressed, and thus is able to act on virtually all body cells. It is produced by macrophages, IFN-alpha have antiviral activities. | IFN-α is known to increase the response of activated T cells, enhance humoral immunity, and promote antigen presentation by antigen-presenting cells. When left unchecked, these responses can be pathological. Several lines of evidence involving patients and mouse models implicate IFN-α, in autoimmune diseases, including systemic lupus erythematosus [47]. Moreover, autoantibodies directed against IFN-α have been reported in systemic lupus erythematosus positively correlating with disease activity [121]. | Bastard et al.[18] showed that all patients tested with autoantibodies against IFN-α2 also had autoantibodies against all 13 IFN-α subtypes, among them anti-IFNα1. |
| Interferon Lambda 3 | IFN-λ3 | Cytokine | cytokine distantly related to type I interferons and the IL-10 family. Plays a critical role in the antiviral host defense, predominantly in the epithelial tissues. Acts as a ligand for the heterodimeric class II cytokine receptor composed of IL10RB and IFNLR1, and receptor engagement leads to the activation of the JAK/STAT signaling pathway resulting in the expression of IFN-stimulated genes (ISG), which mediate the antiviral state. | IFNλs concentrations are increased in the blood and affected tissues of patients with autoimmune rheumatic diseases such as systemic lupus erythematosus, rheumatoid arthritis, Sjögren's syndrome and systemic sclerosis. Increased amounts of IFNλs are also associated with increased disease severity, increased autoantibodies, increased inflammatory markers and specific manifestations in these diseases [122]. | Antibody profiling of a severe COVID-19 cohort identified strongly neutralizing IFN-α and IFN-λ3 autoantibodies in 3 (5.5%) and 2 (3.6%) patients, respectively. IFN-λ3 autoantibodies have been linked to severe forms of COVID-19 [123]. |
| Interleukin-12 | IL-12 (p70) | Cytokine | IL-12p70, a heterodimer composed of p40 and p35 subunits, is produced mainly by macrophages and DCs. Its early production by these cells is important for the induction of NK cells, the elaboration of IFN-γ, and, subsequently, the activation, differentiation, and proliferation of specific Th1 cells. | IL-12 is associated to a wide variety of inflammatory responses associated with autoimmune diseases, including experimental autoimmune encephalomyelitis and collagen-induced arthritis, as well as models of ocular inflammation [124]. Increased IL-12 plasma levels may also be detected in patients with neurological disorders such as multiple sclerosis [125]. On the other hand, high serum IL-12 levels in systemic lupus erythematosus were associated with lupus nephritis [126]. In addition, IL-12 levels reflect RA disease activity [127]. | Antibodies against IL-12p70 have not been reported. However, a study showed higher levels of IL-12 and IL-2 were induced in patients with asymptomatic or mild COVID-19 than in those who with moderate or severe symptoms [3]. In addition, elevated levels of IL-12p70 combined with clinical comorbidities were associated strongly with progression to severe COVID-19 [128]. Recovered COVID-19 patients had elevated levels of IL-12p70, 6 months after initial infection reflecting ongoing chronic inflammation [17]. |
| Liver cytosol type 1 | LC-1 | Autoimmunity | LC-1 can be detected in patients with autoimmune hepatitis type 2 in the presence or absence of Liver-Kidney Microsome (LKM) autoantibodies. | The detection of diagnostic autoantibodies such as anti-LC1 is historically associated with the diagnosis of autoimmune hepatitis [129]. | No data |
| Neuropilin-1 | NRP-1 | Receptor | Cell-surface receptor involved in the development of the cardiovascular system, in angiogenesis, in the formation of certain neuronal circuits and in organogenesis outside the nervous system. There are two neuropilins codified by NRP1 gene, which contain specific protein domains that allow them to participate in several different types of signaling pathways that control cell migration. Neuropilins bind many ligands and various types of co-receptors; they affect cell survival, migration, and attraction. Some of the ligands and co-receptors bound by neuropilins are vascular endothelial growth factor (VEGF) and semaphorin family members. | NRP-1 can be a biomarker of active lupus nephritis, systemic lupus erythematosus patients with active lupus nephritis have increased levels of urinary NRP-1 [130]. Moreover, NRP-1 deposits have been observed in damaged glomerulus areas and positively correlated with clinico-pathological parameters of renal disease in lupus glomerulonephritis [131]. In addition, NRP-1 expression in human salivary gland epithelial cells from patients with Sjögren's syndrome is significantly associated with the release of proangiogenic factors [132]. | Antibodies against NRP-1 have not been reported. However, NRP-1 has been determined as an additional cellular mediator that acts as a co-receptor for SARS-CoV-2 facilitating the entry of this virus into host cells [133]. NRP-1 predominantly binds to the C-terminal of the S1 domain [134]. NRP-1 is implicated in several aspects of a SARS-CoV-2 infection including possible spread through the olfactory bulb and into the central nervous system and increased NRP-1 RNA expression in lungs of severe COVID-19 [135]. |
| Tumor Necrosis Factor Beta - Lymphotoxin Alpha | TNF-β (LT-α) | Cytokine | It is a member of the tumor necrosis factor family, is a cytokine produced by lymphocytes. The protein is highly inducible, secreted, and forms heterotrimers with LT-α which anchor LT-β to the cell surface. This protein also mediates a large variety of inflammatory, immunostimulatory, and antiviral responses, is involved in the formation of secondary lymphoid organs during development and plays a role in apoptosis. | Increased levels of LT-α are found in the serum and synovial tissue of patients with rheumatoid arthritis, and in vitro studies found that LTα-induced proliferation of RA fibroblast-like synoviocytes [136]. In addition, LT-α is increased in the salivary gland secretions and sera of patients with Sjögren's syndrome playing a critical role in the local rather than systemic inflammatory process [137]. | Antibodies against LT-α have not been reported. However, In COVID-19, IL-2, TNF and LT-α are present at lower levels in patients with long duration of viral shedding than from patients with short duration [138]. |
| Angiotensin converting enzyme 2 | ACE2 | Receptor | ACE2 is known to be expressed in various human organs, and its organ- and cell-specific expression suggests that it may play a role in the regulation of cardiovascular and renal function, as well as fertility. ACE cleaves angiotensin I hormone into the vasoconstriction angiotensin II that causes a cascade of hormonal reactions which is part of the body's harmful phase of RAAS, which ultimately leads to an increase in the body's blood pressure. ACE2 has an opposing effect to ACE, degrading angiotensin II into angiotensin, thereby lowering blood pressure. In addition, the encoded protein is a functional receptor for the spike glycoprotein of the human coronavirus HCoV-NL63 and the SARS-CoV and SARS-CoV-2. | RAS axes, formed by ACE, angiotensin II and AT1 receptor and the counter-regulatory one, composed by ACE2, Ang-1-7 and the Mas receptor, modulate inflammation and tissue damage in rheumatoid arthritis [139]. Presence of autoantibodies to ACE2 may be associated with constrictive vasculopathies [140]. | A study showed a significant increase in levels of antibodies against ACE2 in moderate and severe COVID-19 patients relative to patients with mild outcome. The presence of high levels of anti-ACE2 antibodies may lead to further decrease in transmembrane ACE2 activity in the lung and other tissues. Therefore, anti-ACE2 antibodies lead to increase in angiotensin type-1 receptor activity, enhancing the pro-inflammatory responses, and possibly viral internalization and ACE2 shedding and a more severe outcome of the COVID-19 disease [141]. ACE2 autoantibodies develop after SARS-CoV-2 infection and decrease ACE2 activity could lead to an increase in the abundance of Ang II, which causes a proinflammatory state that triggers symptoms of post-acute sequelae after SARS-CoV-2 infection [142]. |
| Branched-Chain Oxo-Acid Dehydrogenase Complex | BCOADC-E2/OGDC-E2/PDC-E2 | Autoimmunity | BCOADC-E2 is the central E2 component of branched chain 2-oxo acid dehydrogenase complex (BCOADC), which is a mitochondrial multienzyme complex involved in the maintenance of the cellular redox state. The target antigens of M2 antibodies have been identified as components of the 2-Oxo-Acid Dehydrogenase Complex, the immune-dominant epitopes being located on the E2 subunits of Pyruvate Dehydrogenase Complex (M2/PDC-E2), Branched-Chain Oxo-Acid Dehydrogenase Complex (M2/ BCOADC-E2) and Oxo-Glutarate Dehydrogenase Complex (M2/OGDC-E2). In most patients, anti-M2 antibodies are directed against various combinations of different epitopes/antigens. Particular cases are however regularly documented where they recognize a single epitope, either linear, conformational or cryptic, on a single subunit. | The target antigens of M2 antibodies have been identified as components of PDC-E2/BCOADC-E2, which react with anti-mitochondrial antibodies in sera of primary biliary cirrhosis patients [143]. | Autoantibodies against PDC-E2 (DLAT) have been observed in patients with brain injury in COVID-19 [24]. |
| Complement component 1q | Complement C1q | Autoimmunity | C1q is a protein that recognizes and regulates the complement cascade.  Binding of C1q to cell receptors can promote phagocytosis, chemotaxis, and procoagulant activity. | C1q deficiency is associated with high prevalence of SLE and the most severe manifestations of the disease. However, complement is also implicated in the effector inflammatory phase of the autoimmune response that characterizes SLE. Moreover, autoantibodies to C1q develop as part of the autoantibody response [144]. | SARS-CoV-2 envelope proteins activate the lectin pathway through binding to mannose-binding lectin (MBL) or the classical pathway through SARS-CoV-2-specific antibodies and C1q [145]. |
| Dense Fine Speckle, 70 Kd | DFS70 | Autoimmunity | Dense Fine Speckled protein of 70 kd molecular weight | Anti-DFS70 antibodies are a sub-group of anti-nuclear antibodies (ANA) that show a fine dense speckled pattern (DFS) by indirect immunofluorescence. This antibody is a useful biological marker in the discrimination of individuals with positive ANA that do not progress to systemic autoimmune disease (SID). This is based on the observation that these antibodies are more common in healthy individuals than in patients with SID, and that healthy individuals with positive anti-DFS70 did not develop SIDs after a clinical follow-up of four years or more [146]. | A case of Kikuchi‐Fujimoto disease with heart involvement after COVID-19 infection reported anti-DFS70 antibodies [147]. |
| Double-stranded DNA | dsDNA | Autoimmunity | Double stranded DNA antigen | Anti-double stranded DNA (Anti-dsDNA) antibodies are a group of anti-nuclear antibodies (ANA) the target antigen of which is double stranded DNA. They are highly diagnostic of systemic lupus erythematosus and are implicated in the pathogenesis of lupus nephritis [148]. | dsDNA antibodies were found in patients with systemic lupus erythematosus developed after COVID-19 infection [149]. |
| Glomerular basement membrane | GBM | Autoimmunity | The glomerular basement membrane (GBM) is a thin (250 to 400 nm) meshwork of extracellular matrix proteins that is an integral part of the glomerular filtration barrier. | Anti–glomerular basement membrane (anti-GBM) antibody disease is a rare autoimmune disorder in which circulating antibodies are directed against an antigen normally present in the GBM and alveolar basement membrane, specifically the alpha-3 chain of type IV collagen. The condition is classified as an immune-complex small vessel vasculitis, the clinical syndrome has a spectrum ranging from mild or no renal involvement to rapidly progressive glomerulonephritis [150]. | There is an association between COVID-19 and anti-glomerular basement membrane (anti-GBM) disease, since the disease had appeared only days to weeks after S the acute SARS-CoV-2 infection [151]. |
| Glycyl-tRNA Synthetase (EJ) | GARS | Autoimmunity | This enzyme belongs to the family of ligases, to be specific those forming carbon-oxygen bonds in aminoacyl-tRNA and related compounds. This enzyme participates in glycine, serine and threonine metabolism and aminoacyl-trna biosynthesis. | Glycyl-tRNA synthetase has been shown to be a target of autoantibodies in the human autoimmune diseases, polymyositis and dermatomyositis [152]. | No data |
| Major zymogen granule membrane glycoprotein 2 | GP2 | Receptor | Functions as an intestinal M-cells transcytotic receptor specific of type-I-piliated bacteria that participates to the mucosal immune response toward these bacteria. | Employing recombinant GP2 as the solid phase antigen, have confirmed the presence of IgA and IgG anti-GP2 PABs in Crohn's disease (CrD) patients and revealed an association of anti-GP2 IgA as well as IgG levels with a specific clinical phenotype in CrD. Also, GP2 plays an important role in modulating innate and acquired intestinal immunity [153]. | No data |
| Interferon‐gamma | IFN-γ | Cytokine | Interferon‐gamma (IFN‐γ) is a cytokine that plays an important role in inducing and modulating an array of immune responses. IFN‐γ is primarily secreted by activated T cells and natural killer (NK) cells, and can promote macrophage activation, mediate antiviral and antibacterial immunity, enhance antigen presentation, orchestrate activation of the innate immune system, coordinate lymphocyte–endothelium interaction, regulate Th1/Th2 balance, and control cellular proliferation and apoptosis. | Several studies have found that IFN-γ, plays a pivotal role in the development and severity of systemic autoimmunity, particularly systemic lupus erythematosus. However, the polivalent nature of IFN-γ identifies possible therapeutic targets that, due to the essential contribution of IFN-γ to systemic autoimmunity, could have some benefits [154]. | Higher IFN-γ levels were detected in early COVID-19 infection than healthy populations. Nevertheless, these levels were not maintained after ten days of symptoms. In those with sustained IFN-γ levels, the mortality increased [155]. |
| Interleukin (IL)-1 alpha | IL-1α | Cytokine | Also known as hematopoietin 1 is a cytokine of the interleukin 1 family that in humans is encoded by the IL1A gene, which is produced mainly by activated macrophages. | IL-1α acts as an alarmin in tissue inflammation and it is actively involved in the pathogenesis of inflammatory and autoimmune diseases, affecting only the local microenvironment [156]. | COVID-19 patients showed lower levels of blood IFN-α compared to controls. A significant increase in blood IFN-α was found over the study period in survivors [157]. |
| Interleukin (IL)-1 beta | IL-1β | Cytokine | Also known as leukocytic pyrogen is encoded by the IL1B gene and is produced by activated macrophages as a proprotein, and then enzimatically processed by caspase 1 to its active form. | IL-1β is a regulator of systemic inflammation, as production of IL-1β by infiltrating macrophages and circulating monocytes produces inflammation beyond the local environment and widespread inflammation [156]. | IL-1β which is released by macrophages, may result in the cytokine storm responsible for the most severe forms of SARS-CoV-2 infection, with indicators of hyperactivation of the immune system and overproduction of inflammatory cytokines [158]. |
| Mi-2 | Mi-2 | Autoimmunity | Mi2 is a SWI2/SNF2 type helicase/ATPase domain-containing protein that was first identified as a dermatomyositis-specific autoantigen, and it seems to be responsible for the chromatin remodeling activity of the NuRD complex. | Autoantibodies against Mi-2 are serologic hallmarks of idiopathic inflammatory myopathies, with a diagnostic sensitivity and specificity of approximately 4-18% and 98-100%, respectively, moreover anti-Mi-2 antibodies are highly associated with dermatomyositis being predictive value for this disease [159]. | No data |
| Proteinase 3 | PR3 | Autoimmunity | Human proteinase 3 (PR3) is a multifunctional serine protease, mainly located in the azurophilic granules and on the cell surface of polymorphonuclear leukocytes (PMN) | Anti-neutrophil cytoplasmatic antibodies (ANCA) with specificity for PR3 are characteristic for patients with Wegener's granulomatosis [160]. | There is a case of new onset anti-PR3 ANCA vasculitis in a COVID-19 antibody positive patient, whether this vasculitis was induced by SARS-CoV-2 is uncertain [161]. |
| Ribo Phosphoprotein (P0, P1, P2) | Ribo Phosphoprotein (P0, P1, P2) | Autoimmunity | Ribosomal P0, P1, and P2 proteins, together with the conserved domain of 28 S rRNA, constitute a major part of the GTPase-associated center in eukaryotic ribosomes. | Antibodies against ribo P are found in approximately in 30% of patients with systemic lupus erythematosus, however there are reports that these autoantibodies appear before the clinical onset of lupus [162]. | No data |
| Ribonucleoprotein/Smith | RNP/Sm | Autoimmunity | RNP and Sm are autoantigens referred as extractable nuclear antigens (ENA). Each ENA is composed of 1 or more proteins associated with small nuclear RNA species (snRNP) ranging in size from 80 to approximately 350 nucleotides. Antibodies to ENAs are common in patients with connective tissue diseases, Sjogren syndrome, systemic sclerosis, and polymyositis/dermatomyositis. | Sm antibodies are a specific serum marker for the diagnosis of systemic lupus erythematosus, and a high titer of RNP antibodies in the absence of other antinuclear antibodies (ANA) is highly suggestive of the diagnosis of mixed connective tissue disease. In SLE, RNP antibodies have been associated with a relatively benign disease course with lower incidence of renal and central nervous system involvement [163]. | No data |
| Beta-2 glycoprotein 1 and Apolipoprotein H | ß2-Glycoprotein 1 (Apo H) | Autoimmunity | β2-glycoprotein 1, also known as beta-2 glycoprotein 1 and Apolipoprotein H (Apo-H), is a 38 kDa multifunctional plasma protein that in humans is encoded by the APOH gene. One of its functions is to bind cardiolipin. | Anti-β2-GP1 antibodies are found in both infectious and some systemic autoimmune diseases, as systemic lupus erythematosus. the phospholipid-binding b2-glycoprotein I is considered to be the main autoantigen in anti-phospholipid syndrome, which main characteristic is thrombosis [164]. | β2GPI median levels were reported to be lower in patients with COVID-19 than in controls, these low levels were associated with ventilatory failure [165]. |
| Transforming growth factor β1 | TGFβ1 | Cytokine | Transforming growth factor β1 (TGFB1) is a multifunctional secreted protein that generally regulates immune function, as well as cell survival and migration, through the Sma- and Mad-related proteins (SMAD) signaling pathway. | Through animal models, it was established that TGF-beta deficiency could induce autoimmune disease by lymphocyte activation and differentiation, cell adhesion molecule expression, regulatory T cell function, the expression of MHC molecules and cytokines, and cell apoptosis [166]. | In severe COVID-19, SARS-CoV-2 triggers a chronic immune reaction that is directed by TGF-β [167]. |
| Tissue transglutaminase | tTG/TG2 | Autoimmunity | Tissue transglutaminase is a calcium‐activated enzyme which cross‐links substrate proteins into insoluble, protease‐resistant complexes, potentially initiating NFT formation. | Antibodies to tissue transglutaminase are found in patients with several conditions, including celiac disease, juvenile diabetes, inflammatory bowel disease, and various forms of arthritis [168]. | A study showed that acute COVID-19 and POST-COVID patients have increased levels of anti-tissue transglutaminase, a specific predictor of celiac disease [169]. |
| Troponin I | Troponin I |  | Human troponin I is presented in cardiac muscle tissue by a single isoform with molecular weight 23876 Da and it consists of 209 amino acid residues. Troponin I is a cardiac and skeletal muscle protein family. It is a part of the troponin protein complex, where it binds to actin in thin myofilaments to hold the actin-tropomyosin complex in place. | No data | Several studies have confirmed that higher values of cardiac troponin I in COVID-19 are significant predictors of severe COVID-19 illness, evidencing that SARS-CoV-2 infection may be associated with different degrees of cardiac injury and dysfunction [170]. |
| Vitronectin | VTN or VN | Receptor | Vitronectin (VTN or VN) is a glycoprotein of the hemopexin family which is abundantly found in serum, the extracellular matrix and bone. In humans it is encoded by the VTN gene. Vitronectin binds to integrin alpha-V beta-3 and thus promotes cell adhesion and spreading. It also inhibits the membrane-damaging effect of the terminal cytolytic complement pathway and binds to several serpins (serine protease inhibitors). | It was demonstrated a significant reduction of vitronectin levels in plasma of Myasthenia gravis patients compared to healthy controls, apparently lower plasma values of this glycoprotein could be prognostic of an impaired complement-dependent immune response [171]. | No data |
| Cluster of differentiation 4) | CD4 | Receptor | CD4 is a co-receptor of the T cell receptor (TCR) and assists the latter in communicating with antigen-presenting cells. The TCR complex and CD4 bind to distinct regions of the antigen-presenting MHC class II molecule. | CD4 T cells of rheumatoid arthritis patients have anomalies in intracellular signaling, repertoire and aging. CD4 T cells might be essential mediators in the development of the chronic inflammation in rheumatoid arthritis. Moreover. CD4 T cells are the most abundant type in the synovial infiltrate, leading to joint damage [172]. | It has been reported that CD4+T and CD8+T numbers are reduced in severe COVID-19 patients compared with non-severe [173]. |
| Glycoprotein-210 | Gp210 | Autoimmunity | Nuclear pore glycoprotein-210 (gp210) is an essential trafficking regulator in the eukaryotic nuclear pore complex. Gp-210 anchors the pore complex to the nuclear membrane and protein tagging reveals its primarily located on the luminal side of double layer membrane at the pore. | Anti-glycoprotein-210 antibodies are directed at gp210 and are found within primary biliary cirrhosis (PBC) patients in high frequency. Moreover, it has been recently reported that these autoantibodies are associated with an adverse phenotype and predicted non-response to treatment [174]. | No data |
| Intrinsic factor | IF/GIF | Autoimmunity | Intrinsic factor (IF), also known as gastric intrinsic factor (GIF), is a glycoprotein produced by the parietal cells in humans of the stomach. It is necessary for the absorption of vitamin B12 later on in the distal ileum of the small intestine. In humans, the gastric intrinsic factor protein is encoded by the GIF gene. | The intrinsic factor is the main autoantigen in autoimmune gastritis. There is recent evidence that gastric mucosa of autoimmune gastritis patients has an elevated proportion (20%) of autoreactive activated CD4+ T-cell clones that recognize intrinsic factor [175]. | No data |
| Jo-1 | Jo-1 | Autoimmunity | The Jo-1 antigen is histidyl-transfer ribonucleic acid (t-RNA) synthetase. This enzyme is partially responsible for attaching t-RNA to their cognate rRNA | The most common of these is the anti Jo-1 antibody directed against the antihistidyl–tRNA synthetase. It is detectable in approximately 15% to 30% of myositis patients overall, and is more common in polymyositis [176]. | Anti Jo-1 antibodies were found in acute COVID-19 patients accompanied by other anti-nuclear antibodies [177]. Moreover, a recent study reported the presence of anti-Jo-1 antibodies in PCS, which were anti-correlated with SARS-CoV-2 antibodies [14]. |
| Myeloperoxidase | MPO | Autoimmunity | Myeloperoxidase (MPO) enzyme is found in neutrophil primary granules and monocyte lysosomes. MPO catalyzes the conversion of hydrogen peroxide to hypochlorite and hypochlorous acid. | High levels and activity of MPO are detected in several autoimmune diseases including multiple sclerosis and the joints of rheumatoid arthritis patients. A pathogenic role for MPO in driving autoimmune inflammation was demonstrated using mouse models [178]. | Infiltrating neutrophils, a hallmark of COVID-19, can release myeloperoxidase, which can activate several pathways that lead to elevated cytokines and production of ROS [179]. |
| Nucleosome | Nucleosome | Autoimmunity | A nucleosome is a section of DNA that is wrapped around a core of proteins. Inside the nucleus, DNA forms a complex with proteins called chromatin, which allows the DNA to be condensed into a smaller volume. When the chromatin is extended and viewed under a microscope, the structure resembles beads on a string. Each of these tiny beads is a called a nucleosome and has a diameter of approximately 11 nm. The nucleosome is the fundamental subunit of chromatin. | Nucleosome is an important antigen in the pathophysiology of systemic lupus erythematosus, and anti-nucleosome antibodies have been associated with organ damage. Nucleosomes seem to be stronger immunogenic than native DNA or histones and induce a robust T-helper cell response [180]. | Nucleosomes as well as its citrullinated form have been found elevated in plasma of COVID-19 patients with a severe prognostic compared to healthy controls [181]. |
| Proliferating cell nuclear antigen | PCNA | Autoimmunity | Proliferating cell nuclear antigen (PCNA) is a DNA clamp that acts as a processivity factor for DNA polymerase δ in eukaryotic cells and is essential for replication. PCNA is a homotrimer and achieves its processivity by encircling the DNA, where it acts as a scaffold to recruit proteins involved in DNA replication, DNA repair, chromatin remodeling and epigenetics. | Kaneda et al., reported in a cohort of systemic lupus erythematosus patients 31% of reactivity with at least one component of the PCNA complex, and most reactive sera contained autoantibodies to several components of the complex. Autoantibodies to PCNA complex were less common in patients with other conective tissue diseases [182]. | An in vitro study showed increased ubiquitination in specific regions (K13, K14, K77, K80, K248, and K254) of PCNA in cells infected with SARS-CoV-2 when compared to a control group [183]. |
| PM/Scl100 | PM/Scl100 | Autoimmunity | The PM/Scl antigen is found in the exosome, a complex consisting of 11-16 proteins in the granular part of the nucleoli and in the nucleoplasm. The proteins act as exoribonucleases during the RNA processing. | Antibodies against the PM-Scl complex are associated with polymyositic/systemic sclerosis overlap syndrome. The antibodies are usually associated with positive ANA with nucleolar patterns. Anti-PM-Scl may be directed to different subunits in the PM-Scl complex, usually the 75 kD and 100 kD subunits. Anti-PM-Scl is often associated with Raynaud's phenomenon, arthritis, muscle aches and effects on the skin and lungs [184]. | Anti-PM/Scl-100 antibodies defined the COVID-19 convalescent group, along with anti-Proteinase 3-ANCA, anti-Beta 2-Glycoprotein and anti-Mi-2 [177]. |
| SmD | SmD | Autoimmunity | SmD is a mixture of three nuclear ribonucleoproteins SmD1, SmD2 and SmD3 | SmD is one of the small nuclear ribonucleoproteins frequently targeted by autoantibodies in systemic lupus erythematosus, moreover, it has been classified as the most specific Sm-antigen in systemic lupus erythematosus [185]. | No Data |
| TNF-like weak inducer of apoptosis | TWEAK (CD255) | Cytokine | TNF-like weak inducer of apoptosis (TWEAK) is a type II transmembrane TNF superfamily member with strong homology to TNF in its extracellular portion, though less potent in inducing apoptosis. It has been clustered as CD255. | Imbalance in the regulation of TWEAK in the process of inflammation and immune modulation leads to the development of chronic inflammation and autoimmune diseases like rheumatoid arthritis, systemic lupus erythematosus, neuroinflammation, multiple sclerosis and ischemic stroke [186]. | No Data |
| Beta-2 Glycoprotein I /apolipoprotein H | Beta2GPI | Autoimmunity | beta2GPI is a human plasma protein with a molecular mass of 50 kDa involved in the coagulation pathway, exerting both procoagulant and anticoagulant activities. | Beta-2 glycoprotein I is the principal target of autoantibodies in the antiphospholipid syndrome [187]. | Anti-Beta 2-Glycoprotein antibodies defined the COVID-19 convalescent group, along with anti-Proteinase 3-ANCA, Anti-PM/Scl-100 and anti-Mi-2 [177]. COVID-19 patients showed higher frequency IgM anti-β2GP1 antibodies than pre-pandemic controls [33]. |
| Fibrinogen | Fibrinogen | Immune mediator | Fibrinogen is a plasma protein synthesized by the liver that is cleaved by thrombin to form fibrin. The circulating half-life of plasma fibrinogen is 3 to 5 days. Fibrinogen is an acute phase reactant and may be significantly increased in acute or chronic inflammatory disorders, pregnancy and estrogen therapy. | Beyond the role played in the coagulation and cardiovascular diseases, fibrinogen is a proinflammatory factor in autoimmune and inflammatory diseases (such as rheumatoid arthritis, vasculitis, inflammatory bowel disease, multiple sclerosis [188]. | COVID-19 patients showed abnormal baseline levels of PT, aPTT, D-dimer, and fibrinogen at admission compared to the control group, moreover, COVID-19 patients with acute respiratory distress syndrome showed a significant increase in levels of fibrinogen compared to those without acute respiratory distress syndrome [189]. |
| Interleukin-22 | IL-22 | Cytokine | Interleukin-22 (IL-22) is an α-helical cytokine that belongs to the IL-10 family cytokine, which is produced by T helper (Th) 17 cells, γδ T cells, NKT cells, and newly described innate lymphoid cells (ILCs). | IL-22 plays a critical role in the inflammation and proliferation cascade of systemic lupus erythematosus, rheumatoid arthritis, multiple sclerosis, Sjogren's syndrome and psoriasis [190]. | IL-22 may contribute to the formation of life-threatening edema enriched with mucins and fibrin, seen in SARS-CoV-2 and SARS-CoV patients [191]. |
| Nucleoporin p62 | Nup62 | Autoimmunity | Nucleoporin p62 (p62) is a 520-amino acids protein associated with the nuclear envelope. p62 is synthesized as a soluble cytoplasmic precursor of 61 kDa followed by modification that involve addition of N-acetylglucosamine residues, followed by association with other complex proteins. | Anti-p62 antibodies (AP62A) are found in a primary biliary cirrhosis, and is prognostic for severe disease [192]. | The viral accessory protein NSP9 of SARS-CoV-2 is reported to interact with nucleoporin 62, he overexpression of NSP9 reduces NUP62 expression on the nuclear envelope, contributing to defective formation of the nuclear pore complex [193]. |
| Vimentin | Vimentin | Autoimmunity | Vimentin is a type III intermediate filament (IF) protein that is expressed in mesenchymal cells. | Post-translationally modified and native forms of vimentin are involved in the pathogenesis of many autoimmune diseases: rheumatoid arthritis, sarcoidosis, systemic lupus erythematosus, antiphospholipid syndrome, Crohn's disease, ankylosing spondyloarthritis and idiopathic pulmonary fibrosis. Modifications of the protein lead to the formation of antigenic epitopes and, as a result, to the synthesis of antibodies [194]. | Vimentin is expressed in human endothelial cells, binds to SARS-CoV-2-spike, and expedites SARS-CoV-2 entry. Vimentin could facilitate SARS-CoV-2 infection and contribute to vascular complications associated with COVID-19 [195]. |

*In cases where autoantibodies associated with COVID-19 or PCS have not been reported, the role of the protein is described.

**References**

1 Shao M, Sun X-L, Sun H, *et al.* Clinical Relevance of Autoantibodies against Interleukin-2 in Patients with Systemic Lupus Erythematosus. *Chin Med J (Engl)* 2018;**131**:1520–6. doi:10.4103/0366-6999.235114

2 Tiberio L, Caruso A, Pozzi A, *et al.* The detection and biological activity of human antibodies to IL-2 in normal donors. *Scand J Immunol* 1993;**38**:472–6. doi:10.1111/j.1365-3083.1993.tb02590.x

3 Tjan LH, Furukawa K, Nagano T, *et al.* Early Differences in Cytokine Production by Severity of Coronavirus Disease 2019. *J Infect Dis* 2021;**223**:1145–9. doi:10.1093/infdis/jiab005

4 Jiang Y, Chen L, Shen J, *et al.* The potential role of abnormal angiotensin-converting enzyme 2 expression correlated with immune infiltration after SARS-CoV-2 infection in the prognosis of breast cancer. *Aging (Albany NY)* 2021;**13**:20886–95. doi:10.18632/aging.203418

5 Botello A, Herrán M, Salcedo V, *et al.* Prevalence of latent and overt polyautoimmunity in autoimmune thyroid disease: A systematic review and meta‐analysis. *Clin Endocrinol (Oxf)* 2020;**93**:375–89. doi:10.1111/cen.14304

6 Mateu-Salat M, Urgell E, Chico A. SARS-COV-2 as a trigger for autoimmune disease: report of two cases of Graves’ disease after COVID-19. *J Endocrinol Invest* 2020;**43**:1527–8. doi:10.1007/s40618-020-01366-7

7 Lui DTW, Lee KK, Lee CH, *et al.* Development of Graves’ Disease After SARS-CoV-2 mRNA Vaccination: A Case Report and Literature Review. *Front Public Heal* 2021;**9**. doi:10.3389/fpubh.2021.778964

8 Phetsouphanh C, Darley DR, Wilson DB, *et al.* Immunological dysfunction persists for 8 months following initial mild-to-moderate SARS-CoV-2 infection. *Nat Immunol* Published Online First: January 2022. doi:10.1038/s41590-021-01113-x

9 Plotnikova M, Lozhkov A, Romanovskaya-Romanko E, *et al.* IFN-λ1 Displays Various Levels of Antiviral Activity In Vitro in a Select Panel of RNA Viruses. *Viruses* 2021;**13**:1602. doi:10.3390/v13081602

10 Lakota K, Thallinger GG, Sodin-Semrl S, *et al.* International cohort study of 73 anti-Ku-positive patients: association of p70/p80 anti-Ku antibodies with joint/bone features and differentiation of disease populations by using principal-components analysis. *Arthritis Res Ther* 2012;**14**:R2. doi:10.1186/ar3550

11 Sacchi MC, Tamiazzo S, Lauritano EC, *et al.* Case report of COVID-19 in an elderly patient: could SARS-CoV2 trigger myositis? *Eur Rev Med Pharmacol Sci* 2020;**24**:11960–3. doi:10.26355/eurrev_202011_23857

12 Kattah NH, Kattah MG, Utz PJ. The U1-snRNP complex: structural properties relating to autoimmune pathogenesis in rheumatic diseases. *Immunol Rev* 2010;**233**:126–45. doi:10.1111/j.0105-2896.2009.00863.x

13 Chang SE, Feng A, Meng W, *et al.* New-onset IgG autoantibodies in hospitalized patients with COVID-19. *Nat Commun* 2021;**12**:5417. doi:10.1038/s41467-021-25509-3

14 Su Y, Yuan D, Chen DG, *et al.* Multiple Early Factors Anticipate Post-Acute COVID-19 Sequelae. *Cell* Published Online First: January 2022. doi:10.1016/j.cell.2022.01.014

15 Puel A, Döffinger R, Natividad A, *et al.* Autoantibodies against IL-17A, IL-17F, and IL-22 in patients with chronic mucocutaneous candidiasis and autoimmune polyendocrine syndrome type I. *J Exp Med* 2010;**207**:291–7. doi:10.1084/jem.20091983

16 Shibabaw T. Inflammatory Cytokine: IL-17A Signaling Pathway in Patients Present with COVID-19 and Current Treatment Strategy. *J Inflamm Res* 2020;**Volume 13**:673–80. doi:10.2147/JIR.S278335

17 Ong SWX, Fong S-W, Young BE, *et al.* Persistent Symptoms and Association With Inflammatory Cytokine Signatures in Recovered Coronavirus Disease 2019 Patients. *Open Forum Infect Dis* 2021;**8**. doi:10.1093/ofid/ofab156

18 Bastard P, Rosen LB, Zhang Q, *et al.* Autoantibodies against type I IFNs in patients with life-threatening COVID-19. *Science (80- )* 2020;**370**. doi:10.1126/science.abd4585

19 González-Moreno J, Raya-Cruz M, Losada-Lopez I, *et al.* Rapidly progressive interstitial lung disease due to anti-MDA5 antibodies without skin involvement: a case report and literature review. *Rheumatol Int* 2018;**38**:1293–6. doi:10.1007/s00296-018-3991-7

20 Giannini M, Ohana M, Nespola B, *et al.* Similarities between COVID-19 and anti-MDA5 syndrome: what can we learn for better care? *Eur Respir J* 2020;**56**:2001618. doi:10.1183/13993003.01618-2020

21 Wang G, Wang Q, Wang Y, *et al.* Presence of Anti-MDA5 Antibody and Its Value for the Clinical Assessment in Patients With COVID-19: A Retrospective Cohort Study. *Front Immunol* 2021;**12**. doi:10.3389/fimmu.2021.791348

22 Suzuki S, Nishikawa A, Kuwana M, *et al.* Inflammatory myopathy with anti-signal recognition particle antibodies: case series of 100 patients. *Orphanet J Rare Dis* 2015;**10**:61. doi:10.1186/s13023-015-0277-y

23 Russo K, Hoch S, Dima C, *et al.* Circulating anticentromere CENP-A and CENP-B antibodies in patients with diffuse and limited systemic sclerosis, systemic lupus erythematosus, and rheumatoid arthritis. *J Rheumatol* 2000;**27**:142–8.

24 Needham EJ, Ren AL, Digby RJ, *et al.* Brain Injury in COVID-19 is Associated with Autoinflammation and Autoimmunity. *medRxiv* 2021;:2021.12.03.21266112. doi:10.1101/2021.12.03.21266112

25 Ugolini-Lopes MR, Mantovani E, Bonoldi VLN, *et al.* Anti-collagen type v: a marker of early systemic sclerosis? *Adv Rheumatol* 2019;**59**:19. doi:10.1186/s42358-019-0063-y

26 Hetemäki I, Laakso S, Välimaa H, *et al.* Patients with autoimmune polyendocrine syndrome type 1 have an increased susceptibility to severe herpesvirus infections. *Clin Immunol* 2021;**231**:108851. doi:10.1016/j.clim.2021.108851

27 McKeon A, Tracy JA. GAD65 neurological autoimmunity. *Muscle Nerve* 2017;**56**:15–27. doi:10.1002/mus.25565

28 Valadez-Calderon J, Ordinola Navarro A, Rodriguez-Chavez E, *et al.* Co-expression of anti-NMDAR and anti-GAD65 antibodies. A case of autoimmune encephalitis in a post-COVID-19 patient. *Neurología* Published Online First: October 2021. doi:10.1016/j.nrl.2021.09.003

29 Brown EM. Anti-Parathyroid and Anti-Calcium Sensing Receptor Antibodies in Autoimmune Hypoparathyroidism. *Endocrinol Metab Clin North Am* 2009;**38**:437–45. doi:10.1016/j.ecl.2009.01.001

30 Abobaker A, Alzwi A. The effect of COVID-19 on parathyroid glands. *J Infect Public Health* 2021;**14**:724–5. doi:10.1016/j.jiph.2021.04.002

31 Bauer A, Habior A, Kraszewska E. Detection of anti-SP100 antibodies in primary biliary cirrhosis. Comparison of ELISA and immunofluorescence. *J Immunoassay Immunochem* 2013;**34**:346–55. doi:10.1080/15321819.2012.741088

32 Rodríguez Y, Rojas M, Monsalve DM, *et al.* Latent autoimmune thyroid disease. *J Transl Autoimmun* 2020;**3**:100038. doi:10.1016/j.jtauto.2020.100038

33 Anaya J-M, Monsalve DM, Rojas M, *et al.* Latent rheumatic, thyroid and phospholipid autoimmunity in hospitalized patients with COVID-19. *J Transl Autoimmun* 2021;**4**:100091. doi:10.1016/j.jtauto.2021.100091

34 Lui DTW, Lee CH, Chow WS, *et al.* Long COVID in Patients With Mild to Moderate Disease: Do Thyroid Function and Autoimmunity Play a Role? *Endocr Pract* 2021;**27**:894–902. doi:10.1016/j.eprac.2021.06.016

35 Meager A, Wadhwa M, Dilger P, *et al.* Anti-cytokine autoantibodies in autoimmunity: preponderance of neutralizing autoantibodies against interferon-alpha, interferon-omega and interleukin-12 in patients with thymoma and/or myasthenia gravis. *Clin Exp Immunol* 2003;**132**:128–36. doi:10.1046/j.1365-2249.2003.02113.x

36 Meager A, Visvalingam K, Peterson P, *et al.* Anti-Interferon Autoantibodies in Autoimmune Polyendocrinopathy Syndrome Type 1. *PLoS Med* 2006;**3**:e289. doi:10.1371/journal.pmed.0030289

37 Goncalves D, Mezidi M, Bastard P, *et al.* Antibodies against type I interferon: detection and association with severe clinical outcome in COVID‐19 patients. *Clin Transl Immunol* 2021;**10**. doi:10.1002/cti2.1327

38 Franceschini F, Cavazzana I. Anti-Ro/SSA and La/SSB antibodies. *Autoimmunity* 2005;**38**:55–63. doi:10.1080/08916930400022954

39 Ge Y, Li S, Li S, *et al.* Interstitial lung disease is a major characteristic of anti-KS associated ant-synthetase syndrome. *Ther Adv Chronic Dis* 2020;**11**:204062232096841. doi:10.1177/2040622320968417

40 Jawaid M, Ross Y, Kamran M. PL-7 Antisynthetase Syndrome in Association with Sjögren’s, Systemic Lupus Erythematosus, and Rheumatoid Arthritis. *Case Rep Rheumatol* 2020;**2020**:1–4. doi:10.1155/2020/4736476

41 Blake T, Noureldin B. Anti-PL-7 antisynthetase syndrome presenting as COVID-19. *Rheumatology* 2021;**60**:e252–4. doi:10.1093/rheumatology/keab129

42 Mor F, Izak M, Cohen IR. Identification of Aldolase as a Target Antigen in Alzheimer’s Disease. *J Immunol* 2005;**175**:3439–45. doi:10.4049/jimmunol.175.5.3439

43 Bauer PR, Kalra S, Osborn TG, *et al.* Influence of autoimmune biomarkers on interstitial lung diseases: A tertiary referral center based case-control study. *Respir Med* 2015;**109**:397–405. doi:10.1016/j.rmed.2015.01.011

44 Luis García de Guadiana Romualdo, Mulero MDR, Olivo MH, *et al.* Circulating levels of GDF-15 and calprotectin for prediction of in-hospital mortality in COVID-19 patients: A case series. *J Infect* 2021;**82**:e40–2. doi:10.1016/j.jinf.2020.08.010

45 Nishimura M, Yamaguchi E, Takahashi A, *et al.* Clinical significance of serum anti-GM-CSF autoantibody levels in autoimmune pulmonary alveolar proteinosis. *Biomark Med* 2018;**12**:151–9. doi:10.2217/bmm-2017-0362

46 Lang FM, Lee KM-C, Teijaro JR, *et al.* GM-CSF-based treatments in COVID-19: reconciling opposing therapeutic approaches. *Nat Rev Immunol* 2020;**20**:507–14. doi:10.1038/s41577-020-0357-7

47 Choubey D, Moudgil KD. Interferons in Autoimmune and Inflammatory Diseases: Regulation and Roles. *J Interf Cytokine Res* 2011;**31**:857–65. doi:10.1089/jir.2011.0101

48 Bastard P, Gervais A, Le Voyer T, *et al.* Autoantibodies neutralizing type I IFNs are present in ~4% of uninfected individuals over 70 years old and account for ~20% of COVID-19 deaths. *Sci Immunol* 2021;**6**. doi:10.1126/sciimmunol.abl4340

49 Wu Q, Yang Q, Lourenco E, *et al.* Interferon-lambda1 induces peripheral blood mononuclear cell-derived chemokines secretion in patients with systemic lupus erythematosus: its correlation with disease activity. *Arthritis Res Ther* 2011;**13**:R88. doi:10.1186/ar3363

50 Aschman T, Schaffer S, Biniaris Georgallis SI, *et al.* Interferon Lambda Regulates Cellular and Humoral Immunity in Pristane-Induced Lupus. *Int J Mol Sci* 2021;**22**:11747. doi:10.3390/ijms222111747

51 Wang EY, Mao T, Klein J, *et al.* Diverse functional autoantibodies in patients with COVID-19. *Nature* 2021;**595**:283–8. doi:10.1038/s41586-021-03631-y

52 Tiberti C, Verrienti A, Fiore B, *et al.* IA-2 combined epitope assay: a new, highly sensitive approach to evaluate IA-2 humoral autoimmunity in type 1 diabetes. *Clin Immunol* 2005;**115**:260–7. doi:10.1016/j.clim.2005.01.015

53 Kim SJ, Jeong DG, Jeong SK, *et al.* Crystal Structure of the Major Diabetes Autoantigen Insulinoma-Associated Protein 2 Reveals Distinctive Immune Epitopes. *Diabetes* 2007;**56**:41–8. doi:10.2337/db06-0237

54 Groth S, Vafia K, Recke A, *et al.* Antibodies to the C-terminus of laminin γ1 are present in a distinct subgroup of patients with systemic and cutaneous lupus erythematosus. *Lupus* 2012;**21**:1482–3. doi:10.1177/0961203312460113

55 Florea F, Koch M, Hashimoto T, *et al.* Autoimmunity against laminins. *Clin Immunol* 2016;**170**:39–52. doi:10.1016/j.clim.2016.07.021

56 Dieker J, Berden JH, Bakker M, *et al.* Autoantibodies against Modified Histone Peptides in SLE Patients Are Associated with Disease Activity and Lupus Nephritis. *PLoS One* 2016;**11**:e0165373. doi:10.1371/journal.pone.0165373

57 Pascolini S, Vannini A, Deleonardi G, *et al.* COVID‐19 and Immunological Dysregulation: Can Autoantibodies be Useful? *Clin Transl Sci* 2021;**14**:502–8. doi:10.1111/cts.12908

58 Meloni A, Furcas M, Cetani F, *et al.* Autoantibodies against Type I Interferons as an Additional Diagnostic Criterion for Autoimmune Polyendocrine Syndrome Type I. *J Clin Endocrinol Metab* 2008;**93**:4389–97. doi:10.1210/jc.2008-0935

59 Chasset F, Dayer J-M, Chizzolini C. Type I Interferons in Systemic Autoimmune Diseases: Distinguishing Between Afferent and Efferent Functions for Precision Medicine and Individualized Treatment. *Front Pharmacol* 2021;**12**. doi:10.3389/fphar.2021.633821

60 Raadsen MP, Gharbharan A, Jordans CCE, *et al.* Interferon-α2 Auto-antibodies in Convalescent Plasma Therapy for COVID-19. *J Clin Immunol* Published Online First: November 2021. doi:10.1007/s10875-021-01168-3

61 Jiang J, Zhao M, Chang C, *et al.* Type I Interferons in the Pathogenesis and Treatment of Autoimmune Diseases. *Clin Rev Allergy Immunol* 2020;**59**:248–72. doi:10.1007/s12016-020-08798-2

62 Raijmakers R, Renz M, Wiemann C, *et al.* PM-Scl-75 is the main autoantigen in patients with the polymyositis/scleroderma overlap syndrome. *Arthritis Rheum* 2004;**50**:565–9. doi:10.1002/art.20056

63 De Lorenzo R, Pinal-Fernandez I, Huang W, *et al.* Muscular and extramuscular clinical features of patients with anti-PM/Scl autoantibodies. *Neurology* 2018;**90**:e2068–76. doi:10.1212/WNL.0000000000005638

64 Fineschi S. Case Report: Systemic Sclerosis After Covid-19 Infection. *Front Immunol* 2021;**12**. doi:10.3389/fimmu.2021.686699

65 Tiwari V, Jandu JS BM. Rheumatoid Factor. In: *StatPearls*. 2022.

66 Baimukhamedov C, Barskova T, Matucci-Cerinic M. Arthritis after SARS-CoV-2 infection. *Lancet Rheumatol* 2021;**3**:e324–5. doi:10.1016/S2665-9913(21)00067-9

67 Derksen VFAM, Kissel T, Lamers-Karnebeek FBG, *et al.* Onset of rheumatoid arthritis after COVID-19: coincidence or connected? *Ann Rheum Dis* 2021;**80**:1096–8. doi:10.1136/annrheumdis-2021-219859

68 Roongta R, Chattopadhyay A, Ghosh A. Correspondence on ‘Onset of rheumatoid arthritis after COVID-19: coincidence or connected?’ *Ann Rheum Dis* 2021;:annrheumdis-2021-220479. doi:10.1136/annrheumdis-2021-220479

69 Fiorentino D, Casciola-Rosen L. Autoantibodies to transcription intermediary factor 1 in dermatomyositis shed insight into the cancer-myositis connection. *Arthritis Rheum* 2012;**64**:346–9. doi:10.1002/art.33402

70 Ghirardello A, Borella E, Beggio M, *et al.* Myositis autoantibodies and clinical phenotypes. *Autoimmun Highlights* 2014;**5**:69–75. doi:10.1007/s13317-014-0060-4

71 Masiak A, Kulczycka J, Czuszyńska Z, *et al.* Clinical characteristics of patients with anti-TIF1-γ antibodies. *Reumatologia/Rheumatology* 2016;**1**:14–8. doi:10.5114/reum.2016.58756

72 Megremis S, Walker TDJ, He X, *et al.* Antibodies against immunogenic epitopes with high sequence identity to SARS-CoV-2 in patients with autoimmune dermatomyositis. *Ann Rheum Dis* 2020;**79**:1383–6. doi:10.1136/annrheumdis-2020-217522

73 Selva-O’Callaghan A, Pinal-Fernandez I, Trallero-Araguás E, *et al.* Classification and management of adult inflammatory myopathies. *Lancet Neurol* 2018;**17**:816–28. doi:10.1016/S1474-4422(18)30254-0

74 Cojocaru M, Cojocaru IM, Chicos B. New Insights into Antisynthetase Syndrome. *Maedica (Buchar)* 2016;**11**:130–5.

75 Ittah M, Miceli-Richard C, Eric Gottenberg J-, *et al.* B cell-activating factor of the tumor necrosis factor family (BAFF) is expressed under stimulation by interferon in salivary gland epithelial cells in primary Sjögren’s syndrome. *Arthritis Res Ther* 2006;**8**:R51. doi:10.1186/ar1912

76 Moisini I, Davidson A. BAFF: a local and systemic target in autoimmune diseases. *Clin Exp Immunol* 2009;**158**:155–63. doi:10.1111/j.1365-2249.2009.04007.x

77 Howe HS, Thong BYH, Kong KO, *et al.* Associations of B cell-activating factor (BAFF) and anti-BAFF autoantibodies with disease activity in multi-ethnic Asian systemic lupus erythematosus patients in Singapore. *Clin Exp Immunol* 2017;**189**:298–303. doi:10.1111/cei.12975

78 Schultheiß C, Paschold L, Simnica D, *et al.* Next-Generation Sequencing of T and B Cell Receptor Repertoires from COVID-19 Patients Showed Signatures Associated with Severity of Disease. *Immunity* 2020;**53**:442-455.e4. doi:10.1016/j.immuni.2020.06.024

79 Wen W, Su W, Tang H, *et al.* Immune cell profiling of COVID-19 patients in the recovery stage by single-cell sequencing. *Cell Discov* 2020;**6**:31. doi:10.1038/s41421-020-0168-9

80 Schultz H. From infection to autoimmunity: A new model for induction of ANCA against the bactericidal/permeability increasing protein (BPI). *Autoimmun Rev* 2007;**6**:223–7. doi:10.1016/j.autrev.2006.08.005

81 Paco S, Kalko SG, Jou C, *et al.* Gene Expression Profiling Identifies Molecular Pathways Associated with Collagen VI Deficiency and Provides Novel Therapeutic Targets. *PLoS One* 2013;**8**:e77430. doi:10.1371/journal.pone.0077430

82 Tian G, Li J-L, Wang D-G, *et al.* Targeting IL-10 in Auto-immune Diseases. *Cell Biochem Biophys* 2014;**70**:37–49. doi:10.1007/s12013-014-9903-x

83 Lu L, Zhang H, Dauphars DJ, *et al.* A Potential Role of Interleukin 10 in COVID-19 Pathogenesis. *Trends Immunol* 2021;**42**:3–5. doi:10.1016/j.it.2020.10.012

84 Han H, Ma Q, Li C, *et al.* Profiling serum cytokines in COVID-19 patients reveals IL-6 and IL-10 are disease severity predictors. *Emerg Microbes Infect* 2020;**9**:1123–30. doi:10.1080/22221751.2020.1770129

85 Ishihara K, Hirano T. IL-6 in autoimmune disease and chronic inflammatory proliferative disease. *Cytokine Growth Factor Rev* 2002;**13**:357–68. doi:10.1016/S1359-6101(02)00027-8

86 Kishimoto T. Interleukin-6: discovery of a pleiotropic cytokine. *Arthritis Res Ther* 2006;**8 Suppl 2**:S2. doi:10.1186/ar1916

87 Jones SA, Hunter CA. Is IL-6 a key cytokine target for therapy in COVID-19? *Nat Rev Immunol* 2021;**21**:337–9. doi:10.1038/s41577-021-00553-8

88 Santa Cruz A, Mendes-Frias A, Oliveira AI, *et al.* Interleukin-6 Is a Biomarker for the Development of Fatal Severe Acute Respiratory Syndrome Coronavirus 2 Pneumonia. *Front Immunol* 2021;**12**. doi:10.3389/fimmu.2021.613422

89 Acosta-Ampudia Y, Monsalve DM, Rojas M, *et al.* Persistent Autoimmune Activation and Proinflammatory State in Post-COVID Syndrome. *medRxiv* Published Online First: 2021. doi:10.1101/2021.11.17.21266457

90 Koralnik IJ, Tyler KL. COVID-19: A Global Threat to the Nervous System. *Ann Neurol* 2020;**88**:1–11. doi:10.1002/ana.25807

91 Lorkiewicz P, Waszkiewicz N. Biomarkers of Post-COVID Depression. *J Clin Med* 2021;**10**:4142. doi:10.3390/jcm10184142

92 Műzes G. Changes of the cytokine profile in inflammatory bowel diseases. *World J Gastroenterol* 2012;**18**:5848. doi:10.3748/wjg.v18.i41.5848

93 Wéra O, Lancellotti P, Oury C. The Dual Role of Neutrophils in Inflammatory Bowel Diseases. *J Clin Med* 2016;**5**:118. doi:10.3390/jcm5120118

94 Cascão R, Moura RA, Perpétuo I, *et al.* Identification of a cytokine network sustaining neutrophil and Th17 activation in untreated early rheumatoid arthritis. *Arthritis Res Ther* 2010;**12**:R196. doi:10.1186/ar3168

95 Li L, Li J, Gao M, *et al.* Interleukin-8 as a Biomarker for Disease Prognosis of Coronavirus Disease-2019 Patients. *Front Immunol* 2021;**11**. doi:10.3389/fimmu.2020.602395

96 Ma A, Zhang L, Ye X, *et al.* High Levels of Circulating IL-8 and Soluble IL-2R Are Associated With Prolonged Illness in Patients With Severe COVID-19. *Front Immunol* 2021;**12**. doi:10.3389/fimmu.2021.626235

97 Li H, Zhang J, Fang C, *et al.* The prognostic value of IL-8 for the death of severe or critical patients with COVID-19. *Medicine (Baltimore)* 2021;**100**:e23656. doi:10.1097/MD.0000000000023656

98 Tomizawa M, Shinozaki F, Fugo K, *et al.* Anti-mitochondrial M2 antibody-positive autoimmune hepatitis. *Exp Ther Med* 2015;**10**:1419–22. doi:10.3892/etm.2015.2694

99 Becker Y, Loignon R-C, Julien A-S, *et al.* Anti-mitochondrial autoantibodies in systemic lupus erythematosus and their association with disease manifestations. *Sci Rep* 2019;**9**:4530. doi:10.1038/s41598-019-40900-3

100 Bartoli A, Gitto S, Sighinolfi P, *et al.* Primary biliary cholangitis associated with SARS-CoV-2 infection. *J Hepatol* 2021;**74**:1245–6. doi:10.1016/j.jhep.2021.02.006

101 Kalluri M, Sahn SA, Oddis C V., *et al.* Clinical Profile of Anti-PL-12 Autoantibody. *Chest* 2009;**135**:1550–6. doi:10.1378/chest.08-2233

102 Yoshimi R, Ueda A, Ozato K, *et al.* Clinical and Pathological Roles of Ro/SSA Autoantibody System. *Clin Dev Immunol* 2012;**2012**:1–12. doi:10.1155/2012/606195

103 Fujii H, Tsuji T, Yuba T, *et al.* High levels of anti-SSA/Ro antibodies in COVID-19 patients with severe respiratory failure: a case-based review. *Clin Rheumatol* 2020;**39**:3171–5. doi:10.1007/s10067-020-05359-y

104 Zhou Y, Han T, Chen J, *et al.* Clinical and Autoimmune Characteristics of Severe and Critical Cases of COVID‐19. *Clin Transl Sci* 2020;**13**:1077–86. doi:10.1111/cts.12805

105 Gracia-Ramos AE, Martin-Nares E, Hernández-Molina G. New Onset of Autoimmune Diseases Following COVID-19 Diagnosis. *Cells* 2021;**10**. doi:10.3390/cells10123592

106 Mahler M, Silverman ED, Schulte-Pelkum J, *et al.* Anti-Scl-70 (topo-I) antibodies in SLE: Myth or reality? *Autoimmun Rev* 2010;**9**:756–60. doi:10.1016/j.autrev.2010.06.005

107 Ugurlu S, Caglar E, Yesim T, *et al.* Anti-Scl-70 Antibodies in Autoimmune Hypothyroidism. *J Int Med Res* 2008;**36**:152–6. doi:10.1177/147323000803600119

108 Gagiannis D, Steinestel J, Hackenbroch C, *et al.* Clinical, Serological, and Histopathological Similarities Between Severe COVID-19 and Acute Exacerbation of Connective Tissue Disease-Associated Interstitial Lung Disease (CTD-ILD). *Front Immunol* 2020;**11**. doi:10.3389/fimmu.2020.587517

109 Ahn SS, Jung SM, Yoo J, *et al.* Anti-Smith antibody is associated with disease activity in patients with new-onset systemic lupus erythematosus. *Rheumatol Int* 2019;**39**:1937–44. doi:10.1007/s00296-019-04445-y

110 Arroyo-Ávila M, Santiago-Casas Y, McGwin G, *et al.* Clinical associations of anti-Smith antibodies in PROFILE: a multi-ethnic lupus cohort. *Clin Rheumatol* 2015;**34**:1217–23. doi:10.1007/s10067-015-2941-y

111 Stascheit F, Hotter B, Hoffmann S, *et al.* Calprotectin as potential novel biomarker in myasthenia gravis. *J Transl Autoimmun* 2021;**4**:100111. doi:10.1016/j.jtauto.2021.100111

112 Kalla R, Kennedy NA, Ventham NT, *et al.* Serum Calprotectin: A Novel Diagnostic and Prognostic Marker in Inflammatory Bowel Diseases. *Am J Gastroenterol* 2016;**111**:1796–805. doi:10.1038/ajg.2016.342

113 Baillet A, Trocmé C, Berthier S, *et al.* Synovial fluid proteomic fingerprint: S100A8, S100A9 and S100A12 proteins discriminate rheumatoid arthritis from other inflammatory joint diseases. *Rheumatology (Oxford)* 2010;**49**:671–82. doi:10.1093/rheumatology/kep452

114 Shokri-Afra H, Alikhani A, Moradipoodeh B, *et al.* Elevated fecal and serum calprotectin in COVID-19 are not consistent with gastrointestinal symptoms. *Sci Rep* 2021;**11**:22001. doi:10.1038/s41598-021-01231-4

115 Mahler M, Meroni P-L, Infantino M, *et al.* Circulating Calprotectin as a Biomarker of COVID-19 Severity. *Expert Rev Clin Immunol* 2021;**17**:431–43. doi:10.1080/1744666X.2021.1905526

116 García de Guadiana-Romualdo L, Rodríguez Rojas C, Morell-García D, *et al.* Circulating levels of calprotectin, a signature of neutrophil activation in prediction of severe respiratory failure in COVID-19 patients: a multicenter, prospective study (CalCov study). *Inflamm Res* 2022;**71**:57–67. doi:10.1007/s00011-021-01516-4

117 Fillit H, Shibata S, Sasaki T, *et al.* Autoantibodies to the Protein Core of Vascular Basement Membrane Heparan Sulfate Proteoglycan in Systemic Lupus Erythematosus. *Autoimmunity* 1993;**14**:243–9. doi:10.3109/08916939309077372

118 Clausen TM, Sandoval DR, Spliid CB, *et al.* SARS-CoV-2 Infection Depends on Cellular Heparan Sulfate and ACE2. *Cell* 2020;**183**:1043-1057.e15. doi:10.1016/j.cell.2020.09.033

119 Zhang Q, Chen CZ, Swaroop M, *et al.* Heparan sulfate assists SARS-CoV-2 in cell entry and can be targeted by approved drugs in vitro. *Cell Discov* 2020;**6**:80. doi:10.1038/s41421-020-00222-5

120 Bermejo‐Jambrina M, Eder J, Kaptein TM, *et al.* Infection and transmission of SARS‐CoV‐2 depend on heparan sulfate proteoglycans. *EMBO J* 2021;**40**. doi:10.15252/embj.2020106765

121 Gupta S, Tatouli IP, Rosen LB, *et al.* Distinct Functions of Autoantibodies Against Interferon in Systemic Lupus Erythematosus: A Comprehensive Analysis of Anticytokine Autoantibodies in Common Rheumatic Diseases. *Arthritis Rheumatol* 2016;**68**:1677–87. doi:10.1002/art.39607

122 Goel RR, Kotenko S V., Kaplan MJ. Interferon lambda in inflammation and autoimmune rheumatic diseases. *Nat Rev Rheumatol* 2021;**17**:349–62. doi:10.1038/s41584-021-00606-1

123 Credle JJ, Gunn J, Sangkhapreecha P, *et al.* Neutralizing IFNL3 Autoantibodies in Severe COVID-19 Identified Using Molecular Indexing of Proteins by Self-Assembly. *bioRxiv* Published Online First: 2021. doi:10.1101/2021.03.02.432977

124 Tait Wojno ED, Hunter CA, Stumhofer JS. The Immunobiology of the Interleukin-12 Family: Room for Discovery. *Immunity* 2019;**50**:851–70. doi:10.1016/j.immuni.2019.03.011

125 Musabak U, Demirkaya S, Genç G, *et al.* Serum Adiponectin, TNF-α, IL-12p70, and IL-13 Levels in Multiple Sclerosis and the Effects of Different Therapy Regimens. *Neuroimmunomodulation* 2011;**18**:57–66. doi:10.1159/000317393

126 Tucci M, Lombardi L, Richards HB, *et al.* Overexpression of interleukin-12 and T helper 1 predominance in lupus nephritis. *Clin Exp Immunol* 2008;**154**:247–54. doi:10.1111/j.1365-2249.2008.03758.x

127 Kim W-U, Min S-Y, Cho M-L, *et al.* The role of IL-12 in inflammatory activity of patients with rheumatoid arthritis (RA). *Clin Exp Immunol* 2002;**119**:175–81. doi:10.1046/j.1365-2249.2000.01095.x

128 Moll-Bernardes R, de Sousa AS, Macedo AVS, *et al.* IL-10 and IL-12 (P70) Levels Predict the Risk of Covid-19 Progression in Hypertensive Patients: Insights From the BRACE-CORONA Trial. *Front Cardiovasc Med* 2021;**8**. doi:10.3389/fcvm.2021.702507

129 Muratori L, Deleonardi G, Lalanne C, *et al.* Autoantibodies in Autoimmune Hepatitis. *Dig Dis* 2015;**33**:65–9. doi:10.1159/000440748

130 Torres-Salido, Sanchis, Solé, *et al.* Urinary Neuropilin-1: A Predictive Biomarker for Renal Outcome in Lupus Nephritis. *Int J Mol Sci* 2019;**20**:4601. doi:10.3390/ijms20184601

131 Vadasz Z, Ben-Izhak O, Bejar J, *et al.* The involvement of immune semaphorins and neuropilin-1 in lupus nephritis. *Lupus* 2011;**20**:1466–73. doi:10.1177/0961203311417034

132 Sisto M, Lisi S, Lofrumento DD, *et al.* Neuropilin-1 is upregulated in Sjögren’s syndrome and contributes to pathological neovascularization. *Histochem Cell Biol* 2012;**137**:669–77. doi:10.1007/s00418-012-0910-y

133 Kyrou I, Randeva HS, Spandidos DA, *et al.* Not only ACE2—the quest for additional host cell mediators of SARS-CoV-2 infection: Neuropilin-1 (NRP1) as a novel SARS-CoV-2 host cell entry mediator implicated in COVID-19. *Signal Transduct Target Ther* 2021;**6**:21. doi:10.1038/s41392-020-00460-9

134 Li Z, Buck M. Neuropilin-1 assists SARS-CoV-2 infection by stimulating the separation of Spike protein S1 and S2. *Biophys J* 2021;**120**:2828–37. doi:10.1016/j.bpj.2021.05.026

135 Mayi BS, Leibowitz JA, Woods AT, *et al.* The role of Neuropilin-1 in COVID-19. *PLOS Pathog* 2021;**17**:e1009153. doi:10.1371/journal.ppat.1009153

136 Hirose T, Fukuma Y, Takeshita A, *et al.* The role of lymphotoxin-α in rheumatoid arthritis. *Inflamm Res* 2018;**67**:495–501. doi:10.1007/s00011-018-1139-6

137 Shen L, Suresh L, Wu J, *et al.* A Role for Lymphotoxin in Primary Sjögren’s Disease. *J Immunol* 2010;**185**:6355–63. doi:10.4049/jimmunol.1001520

138 Yang B, Fan J, Huang J, *et al.* Clinical and molecular characteristics of COVID-19 patients with persistent SARS-CoV-2 infection. *Nat Commun* 2021;**12**:3501. doi:10.1038/s41467-021-23621-y

139 Moreira FRC, de Oliveira TA, Ramos NE, *et al.* The role of renin angiotensin system in the pathophysiology of rheumatoid arthritis. *Mol Biol Rep* 2021;**48**:6619–29. doi:10.1007/s11033-021-06672-8

140 Takahashi Y, Haga S, Ishizaka Y, *et al.* Autoantibodies to angiotensin-converting enzyme 2 in patients with connective tissue diseases. *Arthritis Res Ther* 2010;**12**:R85. doi:10.1186/ar3012

141 Rodriguez-Perez AI, Labandeira CM, Pedrosa MA, *et al.* Autoantibodies against ACE2 and angiotensin type-1 receptors increase severity of COVID-19. *J Autoimmun* 2021;**122**:102683. doi:10.1016/j.jaut.2021.102683

142 Arthur JM, Forrest JC, Boehme KW, *et al.* Development of ACE2 autoantibodies after SARS-CoV-2 infection. *PLoS One* 2021;**16**:e0257016. doi:10.1371/journal.pone.0257016

143 Miyakawa H. Analysis of two major anti-M2 antibodies (anti-PDC-E2/anti-BCOADC-E2) in primary biliary cirrhosis: relationship to titers of immunofluorescent anti-mitochondrial antibody. *Hepatol Res* 2000;**18**:1–9. doi:10.1016/S1386-6346(99)00079-0

144 Walport MJ. Complement and systemic lupus erythematosus. *Arthritis Res Ther* 2002;**4**:S279. doi:10.1186/ar586

145 Afzali B, Noris M, Lambrecht BN, *et al.* The state of complement in COVID-19. *Nat Rev Immunol* Published Online First: December 2021. doi:10.1038/s41577-021-00665-1

146 Aragón C-C, González JD, Posso-Osorio I, *et al.* Anticuerpos anti-DFS70: un nuevo autoanticuerpo útil en la exclusión de patologías autoinmunes. *Rev Colomb Reumatol* 2018;**25**:104–11. doi:10.1016/j.rcreu.2018.01.003

147 Masiak A, Lass A, Kowalski J, *et al.* Self-limiting COVID‐19-associated Kikuchi‐Fujimoto disease with heart involvement: case-based review. *Rheumatol Int* 2022;**42**:341–8. doi:10.1007/s00296-021-05088-8

148 Wang X, Xia Y. Anti-double Stranded DNA Antibodies: Origin, Pathogenicity, and Targeted Therapies. *Front Immunol* 2019;**10**. doi:10.3389/fimmu.2019.01667

149 Zamani B, Moeini Taba S-M, Shayestehpour M. Systemic lupus erythematosus manifestation following COVID-19: a case report. *J Med Case Rep* 2021;**15**:29. doi:10.1186/s13256-020-02582-8

150 McAdoo SP, Pusey CD. Anti-Glomerular Basement Membrane Disease. *Clin J Am Soc Nephrol* 2017;**12**:1162–72. doi:10.2215/CJN.01380217

151 Prema KSJ, Kurien A. Incidence of anti-glomerular basement membrane disease during the COVID-19 pandemic. *Clin Kidney J* 2022;**15**:180–1. doi:10.1093/ckj/sfab204

152 Danoff SK, Casciola-Rosen L. The lung as a possible target for the immune reaction in myositis. *Arthritis Res Ther* 2011;**13**:230. doi:10.1186/ar3347

153 Roggenbuck D, Reinhold D, Werner L, *et al.* Glycoprotein 2 Antibodies in Crohn’s Disease. 2013. 187–208. doi:10.1016/B978-0-12-407681-5.00006-4

154 Pollard KM, Cauvi DM, Toomey CB, *et al.* Interferon-γ and systemic autoimmunity. *Discov Med* 2013;**16**:123–31.

155 Gadotti AC, de Castro Deus M, Telles JP, *et al.* IFN-γ is an independent risk factor associated with mortality in patients with moderate and severe COVID-19 infection. *Virus Res* 2020;**289**:198171. doi:10.1016/j.virusres.2020.198171

156 Cavalli G, Colafrancesco S, Emmi G, *et al.* Interleukin 1α: a comprehensive review on the role of IL-1α in the pathogenesis and treatment of autoimmune and inflammatory diseases. *Autoimmun Rev* 2021;**20**:102763. doi:10.1016/j.autrev.2021.102763

157 Contoli M, Papi A, Tomassetti L, *et al.* Blood Interferon-α Levels and Severity, Outcomes, and Inflammatory Profiles in Hospitalized COVID-19 Patients. *Front Immunol* 2021;**12**. doi:10.3389/fimmu.2021.648004

158 Mardi A, Meidaninikjeh S, Nikfarjam S, *et al.* Interleukin-1 in COVID-19 Infection: Immunopathogenesis and Possible Therapeutic Perspective. *Viral Immunol* 2021;**34**:679–88. doi:10.1089/vim.2021.0071

159 Ghirardello A, Zampieri S, Iaccarino L, *et al.* Anti-Mi-2 antibodies. *Autoimmunity* 2005;**38**:79–83. doi:10.1080/08916930400022681

160 von Vietinghoff S, Schreiber A, Otto B, *et al.* Membrane proteinase 3 and Wegener’s granulomatosis. *Clin Nephrol* 2005;**64**:453–9. doi:10.5414/cnp64453

161 Wintler T, Zherebtsov M, Carmack S, *et al.* Acute PR3-ANCA vasculitis in an asymptomatic COVID-19 teenager. *J Pediatr Surg Case Reports* 2021;**75**:102103. doi:10.1016/j.epsc.2021.102103

162 Heinlen LD, Ritterhouse LL, McClain MT, *et al.* Ribosomal P autoantibodies are present before SLE onset and are directed against non-C-terminal peptides. *J Mol Med* 2010;**88**:719–27. doi:10.1007/s00109-010-0618-1

163 Didier K, Bolko L, Giusti D, *et al.* Autoantibodies Associated With Connective Tissue Diseases: What Meaning for Clinicians? *Front Immunol* 2018;**9**. doi:10.3389/fimmu.2018.00541

164 McDonnell T, Artim-Esen B, Wincup C, *et al.* Antiphospholipid Antibodies to Domain I of Beta-2-Glycoprotein I Show Different Subclass Predominance in Comparison to Antibodies to Whole Beta-2-glycoprotein I. *Front Immunol* 2018;**9**. doi:10.3389/fimmu.2018.02244

165 Serrano M, Espinosa G, Lalueza A, *et al.* Beta‐2‐Glycoprotein‐I Deficiency Could Precipitate an Antiphospholipid Syndrome‐like Prothrombotic Situation in Patients With Coronavirus Disease 2019. *ACR Open Rheumatol* 2021;**3**:267–76. doi:10.1002/acr2.11245

166 Aoki CA, Borchers AT, Li M, *et al.* Transforming growth factor β (TGF-β) and autoimmunity. *Autoimmun Rev* 2005;**4**:450–9. doi:10.1016/j.autrev.2005.03.006

167 Ferreira-Gomes M, Kruglov A, Durek P, *et al.* SARS-CoV-2 in severe COVID-19 induces a TGF-β-dominated chronic immune response that does not target itself. *Nat Commun* 2021;**12**:1961. doi:10.1038/s41467-021-22210-3

168 Caja S, Mäki M, Kaukinen K, *et al.* Antibodies in celiac disease: implications beyond diagnostics. *Cell Mol Immunol* 2011;**8**:103–9. doi:10.1038/cmi.2010.65

169 Lingel H, Meltendorf S, Billing U, *et al.* Unique autoantibody prevalence in long-term recovered SARS-CoV-2-infected individuals. *J Autoimmun* 2021;**122**:102682. doi:10.1016/j.jaut.2021.102682

170 Aggarwal G, Cheruiyot I, Aggarwal S, *et al.* Association of Cardiovascular Disease With Coronavirus Disease 2019 (COVID-19) Severity: A Meta-Analysis. *Curr Probl Cardiol* 2020;**45**:100617. doi:10.1016/j.cpcardiol.2020.100617

171 Lepedda AJ, Deiana GA, Lobina O, *et al.* Plasma vitronectin is reduced in patients with myasthenia gravis: Diagnostic and pathophysiological potential. *J Circ Biomarkers* 2019;**8**:184945441987591. doi:10.1177/1849454419875912

172 Castro-Sánchez P, Roda-Navarro P. Physiology and Pathology of Autoimmune Diseases: Role of CD4+ T cells in Rheumatoid Arthritis. In: *Physiology and Pathology of Immunology*. InTech 2017. doi:10.5772/intechopen.70239

173 Zhang H, Wu T. CD4+T, CD8+T counts and severe COVID-19: A meta-analysis. *J Infect* 2020;**81**:e82–4. doi:10.1016/j.jinf.2020.06.036

174 Haldar D, Janmohamed A, Plant T, *et al.* Antibodies to gp210 and understanding risk in patients with primary biliary cholangitis. *Liver Int* 2021;**41**:535–44. doi:10.1111/liv.14688

175 Troilo A, Grassi A, Petrone L, *et al.* Intrinsic factor recognition promotes T helper 17/T helper 1 autoimmune gastric inflammation in patients with pernicious anemia. *Oncotarget* 2019;**10**:2921–9. doi:10.18632/oncotarget.26874

176 Mileti LM, Strek ME, Niewold TB, *et al.* Clinical Characteristics of Patients With Anti-Jo-1 Antibodies. *JCR J Clin Rheumatol* 2009;**15**:254–5. doi:10.1097/RHU.0b013e3181b0e910

177 Bhadelia N, Belkina AC, Olson A, *et al.* Distinct Autoimmune Antibody Signatures Between Hospitalized Acute COVID-19 Patients, SARS-CoV-2 Convalescent Individuals, and Unexposed Pre-Pandemic Controls. *medRxiv* Published Online First: 2021. doi:10.1101/2021.01.21.21249176

178 Strzepa A, Pritchard KA, Dittel BN. Myeloperoxidase: A new player in autoimmunity. *Cell Immunol* 2017;**317**:1–8. doi:10.1016/j.cellimm.2017.05.002

179 Tang D, Comish P, Kang R. The hallmarks of COVID-19 disease. *PLOS Pathog* 2020;**16**:e1008536. doi:10.1371/journal.ppat.1008536

180 Abdalla MA, Elmofty SA, Elmaghraby AA, *et al.* Anti-nucleosome antibodies in systemic lupus erythematosus patients: Relation to anti-double stranded deoxyribonucleic acid and disease activity. *Egypt Rheumatol* 2018;**40**:29–33. doi:10.1016/j.ejr.2017.05.004

181 Cavalier E, Guiot J, Lechner K, *et al.* Circulating Nucleosomes as Potential Markers to Monitor COVID-19 Disease Progression. *Front Mol Biosci* 2021;**8**. doi:10.3389/fmolb.2021.600881

182 Kaneda K, Takasaki Y, Takeuchi K, *et al.* Autoimmune response to proteins of proliferating cell nuclear antigen multiprotein complexes in patients with connective tissue diseases. *J Rheumatol* 2004;**31**:2142–50.

183 Stukalov A, Girault V, Grass V, *et al.* Multilevel proteomics reveals host perturbations by SARS-CoV-2 and SARS-CoV. *Nature* 2021;**594**:246–52. doi:10.1038/s41586-021-03493-4

184 Hanke K, Brückner CS, Dähnrich C, *et al.* Antibodies against PM/Scl-75 and PM/Scl-100 are independent markers for different subsets of systemic sclerosis patients. *Arthritis Res Ther* 2009;**11**:R22. doi:10.1186/ar2614

185 Mahler M. Sm peptides in differentiation of autoimmune diseases. 2011. 109–28. doi:10.1016/B978-0-12-387025-4.00005-4

186 Bertin D, Stephan D, Khrestchatisky M, *et al.* Is TWEAK a Biomarker for Autoimmune/Chronic Inflammatory Diseases? *Front Immunol* 2013;**4**. doi:10.3389/fimmu.2013.00489

187 Cabrera-Marante O, Rodríguez de Frías E, Serrano M, *et al.* The Weight of IgA Anti-β2glycoprotein I in the Antiphospholipid Syndrome Pathogenesis: Closing the Gap of Seronegative Antiphospholipid Syndrome. *Int J Mol Sci* 2020;**21**:8972. doi:10.3390/ijms21238972

188 Göbel K, Eichler S, Wiendl H, *et al.* The Coagulation Factors Fibrinogen, Thrombin, and Factor XII in Inflammatory Disorders—A Systematic Review. *Front Immunol* 2018;**9**. doi:10.3389/fimmu.2018.01731

189 Di Micco P, Russo V, Carannante N, *et al.* Prognostic Value of Fibrinogen among COVID-19 Patients Admitted to an Emergency Department: An Italian Cohort Study. *J Clin Med* 2020;**9**:4134. doi:10.3390/jcm9124134

190 Pan H-F, Li X-P, Zheng SG, *et al.* Emerging role of interleukin-22 in autoimmune diseases. *Cytokine Growth Factor Rev* 2013;**24**:51–7. doi:10.1016/j.cytogfr.2012.07.002

191 Wu D, Yang XO. TH17 responses in cytokine storm of COVID-19: An emerging target of JAK2 inhibitor Fedratinib. *J Microbiol Immunol Infect* 2020;**53**:368–70. doi:10.1016/j.jmii.2020.03.005

192 Bauer A, Habior A. Detection of Autoantibodies Against Nucleoporin p62 in Sera of Patients With Primary Biliary Cholangitis. *Ann Lab Med* 2019;**39**:291–8. doi:10.3343/alm.2019.39.3.291

193 Makiyama K, Hazawa M, Kobayashi A, *et al.* NSP9 of SARS-CoV-2 attenuates nuclear transport by hampering nucleoporin 62 dynamics and functions in host cells. *Biochem Biophys Res Commun* 2022;**586**:137–42. doi:10.1016/j.bbrc.2021.11.046

194 Musaelyan A, Lapin S, Nazarov V, *et al.* Vimentin as antigenic target in autoimmunity: A comprehensive review. *Autoimmun Rev* 2018;**17**:926–34. doi:10.1016/j.autrev.2018.04.004

195 Amraei R, Xia C, Olejnik J, *et al.* Extracellular vimentin is an attachment factor that facilitates SARS-CoV-2 entry into human endothelial cells. *Proc Natl Acad Sci* 2022;**119**:e2113874119. doi:10.1073/pnas.2113874119
